# Lipid Profile of Plant-Based Milk Alternatives (PBMAs) and Cow’s Milk: A Comparison

**DOI:** 10.1021/acs.jafc.4c03091

**Published:** 2024-08-02

**Authors:** Irene Antunes, Ricardo Bexiga, Carlos Pinto, Helena Gonçalves, Cristina Roseiro, Rui Bessa, Susana Alves, Mário Quaresma

**Affiliations:** † CIISA—Centre for Interdisciplinary Research in Animal Health, Faculty of Veterinary Medicine, University of Lisbon, 1300-477 Lisboa, Portugal; ‡ Associate Laboratory for Animal and Veterinary Sciences (AL4AnimalS), Faculty of Veterinary Medicine, University of Lisbon, 1300-477 Lisboa, Portugal; § Faculdade de Ciências Agrárias e do Ambiente, Universidade dos Açores, 9700-042 Angra do Heroísmo, Açores, Portugal; ∥ Food Technology and Safety Division, National Institute for Agricultural and Veterinary Research (INIAV, IP), Quinta do Marquês, 2780-159 Oeiras, Portugal; ⊥ GeoBioTec—Geobiosciences, Geoengineering e Geobiotechnologies, NOVA School of Science and Technology, Campus de Caparica, 2829-516 Caparica, Portugal

**Keywords:** total lipids, total cholesterol, fatty acid profile, cow’s milk, plant-based milk alternatives

## Abstract

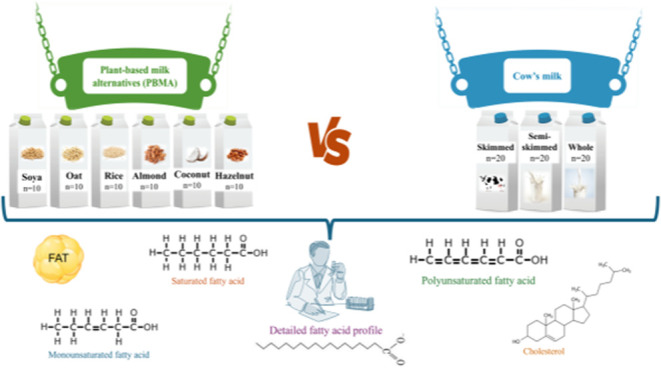

Some consumers are replacing cow’s milk with plant-based milk alternatives (PBMAs). The present study aimed to characterize the lipid profiles of cow’s milk (*n* = 60) and PBMA types (soya, oat, rice, almond, coconut, and hazelnut; *n* = 10 per type). Significant differences were found in the fatty acid (FA) profiles of PBMAs and milk, particularly in FA diversity (15 FAs in PBMAs vs 54 FAs in milk) and the proportion of prime FA groups. The FA profile of coconut was dominated by saturated FAs (SFA), whereas monounsaturated FAs (MUFA) or polyunsaturated FAs (PUFA) were dominant in the remaining PBMA types. Cholesterol was not detected in any PBMA type. The FA profile of milk FAs was dominated by SFA; however, different individual SFA have varying health outcomes. Additionally, milk contains some FA groups with health-promoting properties, such as methyl-branched-chain FAs (BCFA) and conjugated linoleic acid (CLA), both of which are absent in PBMAs.

## 1 Introduction

Dietary fats have important functions in the human body, being the prime energy source and the facilitator of dietary fat-soluble compounds’ absorption (e.g., vitamins).^1^ Moreover, fatty acids (FA) are structural components of cell membranes and regulators of metabolism and gene expression.^1^ Despite these important functions, the role of fat in human nutrition is a matter of debate, and special attention has been given to the intake of saturated fatty acids (SFA) and *trans* FAs. The consumption of these FAs is associated with increased concentrations of blood cholesterol and low-density lipoproteins (LDL).^1^ However, individual SFA have different effects on blood cholesterol, i.e., short-chain (C4:0–C6:0) and medium-chain (C8:0–C10:0) FAs have a slight effect on blood cholesterol concentrations, whereas lauric acid (C12:0), myristic acid (C14:0), and palmitic acid (C16:0) have a high hypercholesterolemic effect.^2,3^

Cow’s milk, hereafter designated as milk, plays a vital role in human nutrition by providing essential nutrients for its growth and development.^4,5^ The link between milk consumption and the development of obesity, type 2 diabetes, cardiovascular disease, osteoporosis, and cancer^6,7^ is a controversial issue, which is mostly sustained by milk’s high SFA and cholesterol contents. Despite that, milk contains some health-beneficial FAs, such as butyric acid, branched-chain FAs (BCFA), and conjugated linoleic acid (CLA) isomers.^8^

According to the FDA (2023),^9^ “plant-based products that are marketed and sold as alternatives to milk are made from nuts (including hazelnuts, walnuts, coconuts, cashews, and almonds), seeds (including sesame, flax, and hemp), rice, oats, or legumes (including soy). The composition of these plant-based milk alternative (PBMA) products, including their nutrient profiles, varies depending on the plant source, processing methods, and added ingredients.” In opposition to milk, PBMAs display no cholesterol, and their FA profile presents high monounsaturated FAs (MUFA) or polyunsaturated FAs (PUFA) contents and low SFA contents, with the exception of coconut PBMA.^7,10^ This information leads consumers to perceive PBMAs as healthier alternatives to milk. Moreover, health-related concerns associated with milk, such as milk protein allergy and lactose intolerance, along with the environmental repercussions of dairy production, are increasing consumers’ concerns, or ethical objections related to animal exploitation (e.g., veganism)^11,12^ lead to an increased demand for PBMAs, particularly in North America, Europe, and East Asia.^13^ In coming years, the PBMA market is expected to grow, reaching around US$19.67 billion in 2023 and US$40.6 billion in 2026.^14,15^

The knowledge regarding PBMA nutritional composition is primarily based on nutrient labeling or nutrient databases^16−22^ and is not sustained by laboratory determinations. Furthermore, considering that FAs play different roles in human health, it is extremely important to determine their lipid profile for consumers’ awareness. The determination and quantification of the FA profile are commonly used to assess the nutritional quality of foods, as well as the FA ratios (polyunsaturated FAs/saturated FAs (P/S), n-6/n-3, and hypocholesterolemic/hypercholesterolemic (h/H)) and the lipid quality indices (atherogenicity index (AI), thrombogenicity index (TI), and peroxidability index (PI)).^23^ Therefore, the present study aims to characterize the lipid profiles of milk and PBMAs through quantification of total cholesterol, total lipid content, and the detailed FA profile, which is sustained by the following two scientific hypotheses: (1) different PBMA types present similar lipid profiles and (2) the lipid profiles of PBMAs and milk from different fat classes show no differences.

## 2 Material and Methods

### 2.1 Sampling and Sample Processing

Milk samples from different fat classes, skimmed (*n* = 20), semi-skimmed (*n* = 20), and whole (*n* = 20), were purchased from the Portuguese market, considering the most representative brands. Plant-based milk alternatives (PBMAs) were also acquired from the Portuguese market, selecting the most representative brands and including six different PBMA types: soya, oat, rice, almond, coconut, and hazelnut (*n* = 10 for each PBMA type). After the samples’ acquisition, they were transported refrigerated to the laboratory, where the content of each package was divided into small containers (properly labeled) and stored in a freezer (−20 °C) for later analysis.

### 2.2 Determination of Total Lipid Content

The total lipid content was determined according to the methodology previously described.^24^ Briefly, approximately 0.5 g of the lyophilized sample was weighed, and 20 mL of the Folch solution (2:1 chloroform/methanol, v/v) was added. The mixture was mixed with a polytron for 1 min, and then it was filtered in a vacuum filter funnel. The filtrate was transferred to a separatory funnel, where 5 mL of sodium chloride (NaCl) solution (0.73% w/v NaCl in distilled water) was added. It was stirred and allowed to stand overnight to separate the phases. On the following day, the lower phase was moved to a previously weighed and identified flask. The contents of the flask were evaporated on a rotary evaporator at 45 °C, and the flask was placed for 1 h in an oven at 100 °C. After this time, the flask was placed in a desiccator to cool and then weighed. Afterward, calculations for the determination of total lipids were performed.

### 2.3 Determination of Total Cholesterol Content

Total cholesterol content was analyzed according to the methodology previously described.^25^ Briefly, 5 mL of each sample was subject to saponification in a water bath at 80 °C for 15 min with 20 mL of saponification solution (11% w/v potassium hydroxide (KOH) in a mixture of 55% v/v absolute ethanol and 45% v/v distilled water) and 0.6 g of ascorbic acid. After this time, the tubes were cooled with tap water for 1 min. Then, 6 mL of distilled water and 12 mL of *n*-hexane with butylated hydroxytoluene (BHT; 25 μg/mL) were added and vortexed for 2 min. The tubes were centrifuged at 1500*g* for 5 min. The top layer was filtered over anhydrous sodium sulfate and filtered again with a 0.45 μm hydrophobic acrodisc (Filter lab, Barcelona, Spain). The filtrate was injected into the high-performance liquid chromatography (HPLC) system. The quantification of cholesterol was performed by normal-phase HPLC (column Zorbax Rx Sil, 4.6 mm ID × 250 mm, 5 μm particle size, Agilent Technologies Inc., Palo Alto, CA) coupled with a ultraviolet–visible (UV–vis) photodiode detector (cholesterol quantification at 202 nm), and the contents were calculated based on the external standard technique. The cholesterol standard was purchased from Sigma Chemical Co. (St. Louis, MO).

### 2.4 Determination of the Fatty Acid Profile

The determination of the fatty acid (FA) profile was performed as previously described.^26^ Briefly, 100 mg (or 200 mg in both semi-skimmed and skimmed milk) of the lyophilized sample was weighed into a test tube, and 2 mL of the internal standard (C19:0; 1 mg/L; Supelco Inc., Bellefonte, PA) and 0.2 mL of 2 M KOH in methanol were added, vortexed for 3 min, and left to stand for 15 min at room temperature. Then, 1 mL of 1.25 M hydrochloric acid (HCl) in methanol was added and vortexed for 10 s. The test tubes were incubated in a water bath at 50 °C for 10 min and allowed to cool at room temperature. At the end of the reaction, 2 mL of Milli-Q water and 1 mL of *n-*hexane were added, vortexed (10 s), and centrifuged for 5 min. The supernatant was collected into a new tube, which already contained 0.5 g of anhydrous sodium sulfate, vortexed (30 s), and centrifuged for 5 min. The *n*-hexane phase was withdrawn with a Pasteur pipet into a GC vial, which was tightly capped and stored in the freezer. FA methyl esters were analyzed by gas chromatography with flame ionization detection (GC-FID) using a Shimadzu GC 2010-Plus (Shimadzu, Kyoto, Japan) equipped with an SP-2560 (100 m × 0.25 mm, 0.20 μm film thickness, Supelco, Bellefonte, PA) capillary column. The chromatographic conditions were as follows: injector and detector temperatures were set at 220 and 250 °C, respectively; helium was used as the carrier gas at 1 mL/min constant flow; the initial oven temperature of 50 °C was held for 1 min, increased at 50 °C/min to 150 °C and held for 20 min, increased at 1 °C/min to 190 °C, and then increased at 2 °C/min to 220 °C and held for 30 min. Identification of FA methyl esters was achieved by a comparison of their retention times with those of commercial standard mixtures (FAME mix 37 components; Supelco Inc., Bellefonte, PA). FAs were expressed as the percentage of the sum of detected FAs (g/100 g of total FAs).

#### 2.4.1 Fatty Acid Ratios and Lipid Quality Indices

The polyunsaturated FAs and saturated FAs ratio (P/S) and the n-6 PUFA family and n-3 PUFA family ratio (n-6/n-3) were calculated as previously established by the British Department of Health (1994).^27^
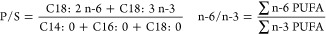
The hypocholesterolemic/hypercholesterolemic ratio (h/H) was calculated using the equation previously proposed by Santos-Silva et al.^28^
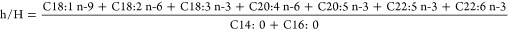
The indices of atherogenicity (AI) and thrombogenicity (TI) were estimated as proposed by Ulbricht and Southgate.^29^
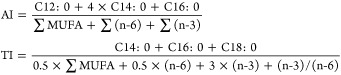
The peroxidability index (PI) was calculated according to the equation previously proposed by Arakawa and Sagai.^30^
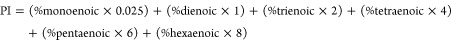


### 2.5 Statistical Analysis

The statistical analysis was carried out using the MIXED procedure of SAS (SAS Inst., Cary, NC) version 9.4. To compare the lipid profile of milk from different fat classes and PBMAs, a statistical model considering the beverage type as a single fixed effect was used, and the different samples of each beverage type were considered as the experimental unit. Multiple comparisons of least-squares means were determined using the PDIFF with the Tukey–Kramer adjustment options of SAS. When needed, the variance heterogeneity was accommodated in the model, using the “Group” option in the “repeated” statement, as described by Milliken and Johnson.^31^ Finally, to describe the variability of data in two dimensions, we performed a principal component analysis (PCA) through the PRINCOMP procedure of SAS. The PCA was applied to 120 samples and 66 variables to describe the variability of the data with two principal components. The principal components’ eigenvalues, loadings of the variables, and the principal components’ loadings of samples are presented in the Supporting Information. In all calculations performed, a density of 1 g/mL was assumed for all beverages.

## 3 Results

### 3.1 Total Lipids

The total lipid (TL) contents are depicted in Table 1. Whole milk displayed the significantly (*P* < 0.05) highest content of TL (3.5 g/100 g), followed by semi-skimmed milk and hazelnut PBMA (averaging 1.6 g/100 g). The remaining PBMA types presented TL contents between 0.6 and 1.08 g/100 g, with significant (*P* < 0.05) differences between them, whereas skimmed milk had the lowest TL content (*P* < 0.05; 0.2 g/100 g).

**Table 1:** Total Lipids (Expressed as g/100 g), Total Cholesterol (Expressed as mg/100 g), Sums (Expressed as % of Total Fatty Acids), Ratios, and Lipid Quality Indices of Cow’s Milk from Different Fat Classes and Different Types of Plant-Based Milk Alternatives (Presented as Mean ± Standard Error)

	cow’s milk			plant-based milk alternatives						
	S^a^	S–S^b^	W^c^	soya	oat	rice	almond	coconut	hazelnut	
*n*	20	20	20	10	10	10	10	10	10	*P*
total lipids	0.2 ± 0.02^f^	1.6 ± 0.04^b^	3.5 ± 0.04^a^	0.7 ± 0.04^e^	0.8 ± 0.08^d,e^	0.6 ± 0.06^e^	1.0 ± 0.03^c^	1.08 ± 0.08^c,d^	1.5 ± 0.03^b^	<0.001
TCHR^d^	3.8 ± 0.76^b^	5.0 ± 0.70^b^	8.9 ± 0.72^a^							<0.001
SFAs^e^	69.6 ± 0.67^b^	71.2 ± 0.90^b^	71.1 ± 0.90^b^	15.4 ± 0.95^c^	16.3 ± 1.27^c^	12.3 ± 1.27^c^	11.5 ± 0.95^c^	79.1 ± 1.27^a^	18.3 ± 5.10^c^	<0.001
MUFAs^f^	25.7 ± 0.71^d,e^	25.5 ± 0.71^d,e^	25.9 ± 0.71^d,e^	22.2 ± 1.00^e^	36.6 ± 1.12^c^	28.2 ± 1.29^d^	67.0 ± 1.05^b^	14.5 ± 1.05^f^	79.8 ± 1.19^a^	<0.001
PUFAs^g^	4.7 ± 0.42^e^	3.4 ± 1.00^e,f^	3.0 ± 0.13^f^	62.4 ± 0.59^a^	46.7 ± 1.41^b^	62.2 ± 1.69^a^	22.8 ± 0.62^c^	5.73 ± 1.87^d,e,f^	12.0 ± 0.71^d^	<0.001
n-6 PUFAs^h^	3.7 ± 0.15^e^	2.4 ± 0.15^f^	2.1 ± 0.15^f^	56.6 ± 1.45^a^	46.0 ± 1.45^b^	61.9 ± 1.73^a^	22.6 ± 1.52^c^	5.54 ± 1.45^d,e,f^	11.9 ± 1.73^d^	<0.001
n-3 PUFAs^i^	0.3 ± 0.05^c^	0.3 ± 0.05^c,d^	0.3 ± 0.05^c,d^	7.3 ± 0.08^a^	1.3 ± 0.10^b^	0.2 ± 0.09^c,d^	0.03 ± 0.077^d^	0.32 ± 0.09^c,d^	0.4 ± 0.13^c,d^	<0.001
P/S^j^	0.06 ± 0.147^d^	0.04 ± 0.147^d^	0.04 ± 0.147^d^	4.3 ± 0.21^a^	3.2 ± 0.36^a,b^	3.9 ± 0.21^a^	2.4 ± 0.21^b^	0.1 ± 0.21^d^	1.4 ± 0.21^c^	<0.001
n-6/n-3^k^	18.0 ± 14.89^d^	8.1 ± 0.40^d^	7.7 ± 0.40^d^	7.6 ± 0.17^d^	127.7 ± 29.78^b,c^	255.3 ± 27.18^b^	678.5 ± 23.54^a^	20.6 ± 38.44^c,d^	129.8 ± 23.54^c^	<0.001
h/H^l^	0.6 ± 0.02^d^	0.5 ± 0.03^d,e^	0.4 ± 0.03^e^	7.8 ± 0.44^c^	6.6 ± 0.44^c^	11.1 ± 0.53^b^	11.8 ± 0.47^b^	0.3 ± 0.44^d,e^	15.2 ± 0.53^a^	<0.001
AI^m^	2.7 ± 0.07^b^	3.2 ± 0.43^b^	3.2 ± 0.43^b^	0.1 ± 0.01^c,d^	0.2 ± 0.02^c^	0.1 ± 0.02^c,d^	0.1 ± 0.01^d^	11.4 ± 0.61^a^	0.2 ± 0.10^c,d^	<0.001
TI^n^	3.4 ± 0.20^b^	3.8 ± 0.31^b^	3.8 ± 0.31^b^	0.3 ± 0.28^c^	0.4 ± 0.28^c^	0.3 ± 0.28^c^	0.3 ± 0.28^c^	7.8 ± 0.28^a^	0.5 ± 0.28^c^	<0.001
PI^o^	6.5 ± 0.71^e^	4.2 ± 0.83^e,f^	3.7 ± 0.15^f^	68.9 ± 1.18^a^	48.5 ± 1.36^b^	63.1 ± 1.41^a^	24.5 ± 1.43^c^	6.9 ± 1.06^e,f^	14.0 ± 1.63^d^	<0.001

a Skimmed milk.

b Semi-skimmed milk.

c Whole milk.

d Total cholesterol content.

e Sum of saturated fatty acids.

f Sum of monosaturated fatty acids.

g Sum of polyunsaturated fatty acids.

h n-6 PUFA family.

i n-3 PUFA family.

j Polyunsaturated fatty acids and saturated fatty acids ratio.

k n-6 PUFA family and n-3 PUFA family ratio.

l Hypocholesterolemic/hypercholesterolemic ratio.

m Atherogenicity index.

n Thrombogenicity index.

o Peroxidability index.

### 3.2 Total Cholesterol

Whole milk displayed (*P* < 0.05) higher total cholesterol content (8.9 mg/100 g) than both skimmed and semi-skimmed milk (averaging 4.4 mg/100 g; Table 1). In contrast, total cholesterol was not quantified in any PBMA types.

### 3.3 Fatty Acid Partial Sums, Fatty Acid Ratios, and Lipid Quality Indices

The coconut PBMA presented the highest total saturated fatty acids (SFA) content (*P* < 0.001; 79.1% of total fatty acids (TFAs)) among all beverages in comparison, surpassing even the milk groups, which had an intermediary total SFA content (averaging 70.6% of TFAs). Conversely, the remaining PBMA types presented the lowest total SFA content (*P* > 0.05, averaging 14.7% of TFAs; Table 1).

In total monounsaturated fatty acids (MUFA) content, hazelnut PBMA displayed the significantly (*P* < 0.05) highest value (79.8% of TFAs), followed by almond PBMA, which had a significantly (*P* < 0.05) lower content than the former (67% of TFAs). Coconut PBMA showed the lowest total MUFA content (14.5% of TFAs), whereas the remaining PBMA types and milk groups presented total MUFA contents between 22.2 and 36.6% of TFAs.

The soya and rice PBMAs displayed the highest total polyunsaturated fatty acids (PUFA) content, averaging 62.3% of TFAs. On the other hand, the coconut PBMA displayed the lowest total PUFA content (5.7% TFA), not significantly (*P* > 0.05) different from the milk groups and hazelnut PBMA. However, in milk groups, the total PUFA content was significantly (*P* < 0.05) higher when the TL was lower. Oat and almond PBMAs displayed intermediated contents, with a significant (*P* < 0.05) difference between them.

Regarding PUFA families (n-3 and n-6 PUFA), the soya PBMA displayed the highest total n-3 PUFA content (*P* < 0.05; 7.3% of TFAs), followed by oat PBMA, which had a significantly (*P* < 0.05) lower content (1.3% of TFAs). The remaining PBMA types and milk groups presented significantly lower total n-3 PUFA contents, with significant (*P* < 0.05) differences among them. In the total n-6 PUFA, soya and rice PBMAs displayed the highest contents (averaging 59.3% of TFAs), followed by oat PBMA (*P* < 0.05; 46% of TFAs) and almond PBMA (*P* < 0.05; 22.6% of TFAs). Hazelnut and coconut were the PBMA types that showed the lowest total n-6 PUFA contents, averaging 8.7% of TFAs. Furthermore, the total n-6 PUFA content of coconut PBMA did not differ significantly (*P* > 0.05) from the contents presented by milk groups. Among milk groups, skimmed milk had a significantly (*P* < 0.05) higher total n-6 PUFA content (3.7% of TFAs) compared to both semi-skimmed and whole milk, which did not present significant (*P* > 0.05) differences between them (averaging 2.3% of TFAs).

Regarding fatty acid (FA) ratios, soya and rice PBMAs displayed the highest values (*P* < 0.001) of the P/S ratio (averaging 4.1); however, it was not significantly (*P* > 0.05) different from the value presented by oat PBMA (3.2). Almond and hazelnut PBMAs presented intermediated values, but significantly (*P* < 0.05) different between them, while coconut PBMA and the milk groups displayed the lowest values, with no significant (*P* > 0.05) differences among them (averaging 0.05). Moreover, almond PBMA presented the highest value of the n-6/n-3 ratio (*P* < 0.05; 678.5), while the remaining PBMA types showed significantly (*P* < 0.05) lower values. The milk groups and soya PBMA had the lowest values (*P* < 0.05; averaging 13.6), which were not significantly (*P* > 0.05) different from the coconut PBMA value (20.6). Lastly, regarding the h/H ratio, hazelnut PBMA showed the significantly (*P* < 0.05) highest value (15.2). Soya, oat, rice, and almond PBMAs displayed intermediated values with significant (*P* < 0.05) differences among them. Coconut PBMA and semi-skimmed milk had lower values (*P* < 0.05; averaging 0.4), which were not significantly (*P* > 0.05) different from the values shown by both skimmed and whole milk.

Regarding lipid quality indices, coconut PBMA had significantly (*P* < 0.001) higher values of the atherogenicity index (AI) and thrombogenicity index (TI; 11.4 and 7.8, respectively), followed by the milk groups (*P* > 0.05; averaging 3.0 and 3.7, respectively). The remaining PBMA types showed lower values, with significant (*P* < 0.05) differences in AI but not in TI (*P* > 0.05; averaging 0.34). On the other hand, soya and rice PBMAs displayed the significantly (*P* < 0.05) highest PI values (averaging 66), followed by oat and almond PBMAs with a lower PI value (*P* < 0.05; 48.5 and 24.5, respectively). The other PBMA types, as well as the milk groups, presented even lower PI values, with significant (*P* < 0.05) differences among them.

### 3.4 Fatty Acid Profile

There were considerable differences between the FA profile of milk groups and the FA profile of PBMAs. The former presented 54 individual FAs (24 were SFA, 20 were MUFA, and 10 were PUFA), whereas the latter presented only 15 individual FAs (eight were SFA, four were MUFA, and three were PUFA; Tables 2 and 3).

**Table 2:** Saturated Fatty Acid Profile (Expressed as % of Total Fatty Acids) of Cow’s Milk with Different Fat Classes and Different Types of Plant-Based Milk Alternatives (Presented as Mean ± Standard Error)

	cow’s milk			plant-based milk alternatives						
	S^a^	S–S^b^	W^c^	soya	oat	rice	almond	coconut	hazelnut	
*n*	20	20	20	20	10	10	10	10	10	*P*
C4:0	2.8 ± 0.31	2.5 ± 0.31	2.3 ± 0.31							0.514
C6:0	1.7 ± 0.05^b^	1.9 ± 0.06^a^	2.0 ± 0.05^a^							0.006
C8:0	1.3 ± 0.02	1.3 ± 0.02	1.3 ± 0.02							0.964
C10:0	2.8 ± 0.07^b^	3.2 ± 0.07^a^	3.2 ± 0.05^a^							0.0002
C11:0^d^	0.39 ± 0.043	0.44 ± 0.014	0.42 ± 0.014							0.516
C12:0	3.4 ± 0.08^b^	3.9 ± 0.08^a^	3.8 ± 0.08^a^							<0.001
C13:0 *anteiso*	0.19 ± 0.037	0.10 ± 0.005	0.11 ± 0.005							0.050
C13:0	0.14 ± 0.028	0.13 ± 0.010	0.12 ± 0.005							0.623
C14:0 *iso*	0.15 ± 0.039	0.11 ± 0.008	0.11 ± 0.012							0.566
C14:0	11.1 ± 0.15^c^	11.8 ± 0.15^b^	11.8 ± 0.15^b^	0.7 ± 0.08^d^	0.2 ± 0.02^e^	0.2 ± 0.02^e^	0.1 ± 0.02^f^	50.6 ± 1.65^a^	0.1 ± 0.01^f^	<0.001
C15:0 *iso*	0.33 ± 0.129	0.22 ± 0.010	0.22 ± 0.010							0.719
C15:0 *anteiso*	0.41 ± 0.065	0.46 ± 0.012	0.49 ± 0.032							0.580
C15:0	0.91 ± 0.095	1.07 ± 0.034	1.01 ± 0.034							0.205
C16:0 *iso*	0.24 ± 0.043	0.24 ± 0.011	0.23 ± 0.011							0.764
C16:0	32.1 ± 0.50^a^	33.5 ± 0.71^a^	33.8 ± 0.50^a^	10.4 ± 0.62^c,d^	13.6 ± 1.01^c^	8.1 ± 1.01^d,e^	8.6 ± 0.62^d,e^	21.1 ± 0.62^b^	5.9 ± 0.74^e^	<0.001
C17:0 *iso*	0.17 ± 0.039^b^	0.29 ± 0.039^a,b^	0.32 ± 0.039^a^							0.022
C17:0	0.30 ± 0.050^a^	0.46 ± 0.009^a^	0.44 ± 0.049^a^	0.10 ± 0.017^b^	0.05 ± 0.014^b^	0.04 ± 0.013^b^	0.07 ± 0.012^b^	0.03 ± 0.019^b^	0.09 ± 0.012^b^	<0.001
cyclo-17^e^	0.09 ± 0.014	0.11 ± 0.008	0.11 ± 0.008							0.329
C18:0	10.3 ± 0.31^a^	9.0 ± 0.15^b^	9.0 ± 0.15^b^	4.6 ± 0.22^d^	2.0 ± 0.206^f^	3.1 ± 0.206^e^	2.6 ± 0.206^e,f^	7.3 ± 0.206^c^	2.2 ± 0.246^e,f^	<0.001
C20:0	0.25 ± 0.056	0.14 ± 0.006	0.14 ± 0.009							0.167
C21:0	0.18 ± 0.019^a^	0.05 ± 0.018^b^	0.04 ± 0.018^b^	0.05 ± 0.032^b^	0.03 ± 0.035^b^	0.07 ± 0.032^a,b^	0.03 ± 0.025^b^	0.02 ± 0.045^b^	0.02 ± 0.030^b^	<0.001
C22:0	0.19 ± 0.019^c^	0.04 ± 0.015^d^	0.04 ± 0.015^d^	0.33 ± 0.021^b^	0.41 ± 0.030^b^	0.61 ± 0.021^a^	0.03 ± 0.022^d^	0.07 ± 0.022^d^	0.03 ± 0.025^d^	<0.001
C23:0	0.10 ± 0.012^a^	0.02 ± 0.013^b^	0.02 ± 0.012^b^	0.04 ± 0.026^a,b^	0.03 ± 0.030^a,b^	0.03 ± 0.023^a,b^	0.02 ± 0.026^a,b^		0.08 ± 0.019^a,b^	0.0001
C24:0	0.12 ± 0.025^a^	0.02 ± 0.004^b^	0.02 ± 0.004^b^	0.07 ± 0.054^a,b^	0.12 ± 0.038^a,b^	0.09 ± 0.058^a,b^	0.12 ± 0.048^a,b^	0.08 ± 0.003^a^	0.04 ± 0.054^a,b^	<0.001

a Skimmed milk.

b Semi-skimmed milk.

c Whole milk.

d Coelution of C11:0 and C10:1 *cis-*9.

e 11-Cyclohexyl-11:0.

**Table 3:** Unsaturated Fatty Acid Profile (Expressed as % of Total Fatty Acids) of Cow’s Milk with Different Fat Classes and Different Types of Plant-Based Milk Alternatives (Presented as Mean ± Standard Error)

	cow’s milk			plant-based milk alternatives						
	S^a^	S–S^b^	W^c^	soya	oat	rice	almond	coconut	hazelnut	
*n*	20	20	20	10	10	10	10	10	10	*P*
C12:1	0.14 ± 0.024	0.12 ± 0.009	0.11 ± 0.004							0.212
C14:1 *cis-*9	0.92 ± 0.073^b^	1.11 ± 0.018^a^	1.14 ± 0.023^a^							0.026
C16:1 *cis-*7	0.15 ± 0.030	0.18 ± 0.006	0.18 ± 0.006							0.637
C16:1 *cis-*9	1.71 ± 0.098^a^	1.82 ± 0.085^a^	1.66 ± 0.085^a^	0.06 ± 0.018^e^	0.16 ± 0.012^c,d^	0.13 ± 0.015^d,e^	0.48 ± 0.012^b^		0.20 ± 0.014^c^	<0.001
C17:1 *cis-*9	0.12 ± 0.033	0.21 ± 0.008	0.20 ± 0.005							0.070
C18:1 *cis-*9	19.9 ± 0.72^e^	19.1 ± 0.21^e^	18.6 ± 0.73^e^	20.6 ± 1.01^e^	35.9 ± 1.01^c^	26.7 ± 1.43^d^	65.3 ± 1.07^b^	13.9 ± 1. 01^f^	78.3 ± 1.21^a^	<0.001
C18:1 *cis-*11	0.35 ± 0.039^d^	0.58 ± 0.039^b,c^	0.55 ± 0.039^c^	1.18 ± 0.06^a^	0.71 ± 0.02^b^	0.69 ± 0.02^b^	1.12 ± 0.02^a^	0.17 ± 0.02^e^	1.15 ± 0.07^a^	<0.001
C18:1 *cis-*12	0.40 ± 0.184	0.27 ± 0.007	0.27 ± 0.007							0.702
C18:1 *cis-*13	0.18 ± 0.056	0.05 ± 0.007	0.07 ± 0.056							0.104
C18:1 *cis-*16	0.09 ± 0.027	0.05 ± 0.005	0.05 ± 0.005							0.416
C18:1 *trans*-6^d^	0.15 ± 0.023^b^	0.27 ± 0.022^a^	0.28 ± 0.022^a^							<0.001
C18:1 *trans*-9	0.18 ± 0.053	0.22 ± 0.010	0.23 ± 0.004							0.578
C18:1 *trans*-10	0.26 ± 0.058^b^	0.48 ± 0.018^a^	0.46 ± 0.018^a^							0.005
C18:1 *trans*-11	0.50 ± 0.078^b^	0.80 ± 0.045^a^	0.75 ± 0.045^a^							0.007
C18:1 *trans-*12	0.17 ± 0.025^b^	0.43 ± 0.025^a^	0.47 ± 0.011^a^							<0.001
C18:1 *trans-*15	0.17 ± 0.030	0.22 ± 0.012	0.21 ± 0.006							0.344
C18:1 *trans-*16^e^	0.22 ± 0.047	0.26 ± 0.011	0.40 ± 0.166							0.551
C18:2 *cis-*9,*trans-*11	0.31 ± 0.037	0.39 ± 0.036	0.41 ± 0.036							0.184
C18:2 *cis*,*trans*/*trans*,*cis*^f^	0.32 ± 0.049	0.27 ± 0.049	0.24 ± 0.051							0.475
C18:2 *trans-*9,*cis-1*2	0.10 ± 0.011^a^	0.04 ± 0.011^b^	0.04 ± 0.011^b^							0.0004
C18:2 *trans-*11,*cis-*15	0.10 ± 0.026	0.06 ± 0.006	0.05 ± 0.006							0.195
C18:2 n-6	2.6 ± 0.13^f^	2.0 ± 0.05^g^	1.8 ± 0.13^g^	56.6 ± 1.48^a^	45.9 ± 1.48^b^	61.9 ± 1.77^a^	22.8 ± 1.56^c^	3.7 ± 0.23^e^	11.9 ± 1.77^d^	<0.001
C18:3 n-6	0.15 ± 0.025^a^	0.03 ± 0.003^b^	0.05 ± 0.025^b^							<0.001
C18:3 n-3	0.33 ± 0.028^c^	0.30 ± 0.016^c^	0.28 ± 0.014^c^	7.33 ± 0.244^a^	0.83 ± 0.142^b^	0.24 ± 0.070^c,d^	0.04 ± 0.004^d^	0.58 ± 0.118^b,c^	0.18 ± 0.050^c,d^	<0.001
C19:1	0.10 ± 0.021	0.07 ± 0.005	0.08 ± 0.005							0.184
C20:1	0.26 ± 0.025^a,b^	0.10 ± 0.025^c^	0.10 ± 0.025^c^	0.36 ± 0.036^a^	0.15 ± 0.040^b,c^	0.26 ± 0.036^a,b^	0.09 ± 0.038^c^	0.22 ± 0.043^a,b,c^	0.12 ± 0.043^b,c^	<0.001
C20:2 n-6	0.17 ± 0.050	0.03 ± 0.006	0.04 ± 0.005	0.03 ± 0.010	0.03 ± 0.013	0.01 ± 0.029	0.01 ± 0.029			0.110
C20:3 n-6	0.21 ± 0.043	0.11 ± 0.007	0.11 ± 0.006							0.091
C20:4 n-6	0.51 ± 0.111^a^	0.16 ± 0.007^b^	0.15 ± 0.005^b^							0.003

a Skimmed milk.

b Semi-skimmed milk.

c Whole milk.

d C18:1 *trans*-6/t*rans-*7/*trans*-8 (coelution).

e C18:1 *trans*-16/*cis*-14 (coelution).

f Sums of several isomers with unknown geometry.

Twelve SFA displayed significant (*P* < 0.05) differences in their contents, while the other half showed no significant (*P* > 0.05) differences. C14:0, C16:0, C17:0, C18:0, C21:0, C22:0, C23:0, and C24:0 were detected in both milk groups and PBMA types, whereas the remaining individual FAs were only detected in the milk groups. In the milk groups, both whole and semi-skimmed milk displayed significantly (*P* < 0.05) higher contents of C6:0, C10:0, and C12:0 (averaging 2.0% of TFAs, 3.2% of TFAs, and 3.9% of TFAs, respectively) compared to skimmed milk (1.7% of TFAs, 2.8% of TFAs, and 3.4% of TFAs, respectively). For C17:0 *iso*, whole milk had the significantly (*P* < 0.05) highest content (0.32% of TFAs), while skimmed milk had the lowest content (0.17% of TFAs). Semi-skimmed milk showed an intermediate content (0.29% of TFAs).

For individual FAs common in both PBMA types and milk groups, coconut PBMA displayed the highest (*P* < 0.05) content of C14:0 (50.6% of TFAs), followed by both whole milk and semi-skimmed milk with lower contents (*P* < 0.05; averaging 33.7% of TFAs) and skimmed milk with an even lower content (*P* < 0.05; 32.1% of TFAs). On the other hand, milk groups displayed the highest (*P* < 0.05) contents of C16:0 (averaging 33.1% of TFAs), followed by coconut PBMA with a significantly lower content (*P* < 0.05; 21.1% of TFAs). Furthermore, skimmed milk had a significantly (*P* < 0.05) higher C18:0 content than both semi-skimmed and whole milk (10.3% of TFAs vs 9% of TFAs), followed by coconut PBMA with a significant (*P* < 0.05) lower content (7.3% of TFAs). For the individual FAs mentioned above, the remaining PBMA types presented lower contents, with significant (*P* < 0.05) differences among them.

The milk groups showed higher contents of C17:0 and C21:0 compared to PBMA types. However, there were significant (*P* < 0.05) differences in C21:0 contents among milk groups, and the rice PBMA had a C21:0 content not significantly different (*P* > 0.05) from that of skimmed milk. Furthermore, rice PBMA showed the significantly (*P* < 0.05) highest content of C22:0, followed by oat and soya PBMAs, which had significantly (*P* < 0.05) lower contents (averaging 0.37% of TFAs). Finally, skimmed milk had the significantly (*P* < 0.05) highest contents of C23:0 and C24:0 (0.10% of TFAs and 0.12% of TFAs, respectively), with the latter not being significantly (*P* > 0.05) different from the content in coconut PBMA (0.08% of TFAs). For both individual FAs, the remaining PBMA types showed intermediated contents (averaging 0.03% of TFAs and 0.10% of TFAs, respectively), while both semi-skimmed and whole milk displayed the lowest contents (averaging 0.02% of TFAs and 0.02% of TFAs, respectively).

Regarding the unsaturated individual FAs, out of the 29 FAs quantified, 14 showed significant (*P* < 0.05) differences in their contents, while the others did not show significant (*P* > 0.05) differences. Furthermore, not all 29 FAs were quantified in all groups. C16:1 *cis-*9, C18:1 *cis-*9, C18:1 *cis-*11, C18:2 n-6, C18:3 n-3, C20:1, and C20:2 n-6 were quantified in both milk groups and PBMA types, while the remaining individual FAs were quantified only in the milk groups. Both semi-skimmed and whole milk displayed significantly (*P* < 0.05) higher contents of C14:1 *cis-*9, C18:1 *trans*-6/*trans*-7/*trans*-8, C18:1 *trans-*10, C18:1 *trans-11*, and C18:1 *trans-*12 than skimmed milk. Conversely, skimmed milk had significantly (*P* < 0.05) higher contents of C18:2 *trans-*9,*cis-*12, C18:3 n-6, and C20:4 n-6 than semi-skimmed and whole milk.

Regarding individual FAs common to both PBMA types and the milk groups, PBMAs generally contained significantly (*P* < 0.05) higher levels of individual MUFA and PUFA compared to the milk groups. The exception was C16:1 *cis*-9, where the milk groups exhibited significantly (*P* < 0.05) higher levels, averaging 1.73% of TFAs, while PBMA types displayed significant (*P* < 0.05) lower contents, with significant (*P* < 0.05) differences among them. In terms of C18:1 *cis*-9, hazelnut PBMA had the highest content (78.3% of TFAs), followed by almond, oat, and rice PBMAs with lower contents (*P* < 0.05; 65.3% of TFAs, 35.9% of TFAs, and 26.7% of TFAs, respectively). The milk groups, along with soya PBMA, had a C18:1 *cis*-9 content of 19.6% of TFAs, whereas coconut PBMA had the lowest content of this FA (*P* < 0.05; 13.9% of TFAs). Additionally, soya, almond, and hazelnut PBMAs displayed the highest (*P* < 0.05) C18:1 *cis*-11 contents, averaging 1.15% of TFAs. Conversely, coconut PBMA had the lowest content of this FA (*P* < 0.05; 0.17% of TFAs), while the remaining PBMA types and the milk groups showed intermediate contents, with significant (*P* < 0.05) differences among them. Rice PBMA had the highest C18:2 n-6 content (61.9% of TFAs), though not significantly different (*P* > 0.05) from soya PBMA (59.2% of TFAs). Soya PBMA also had the highest C18:3 n-3 and C20:1 contents (*P* < 0.05, averaging 7.33% of TFAs and 0.36% of TFAs, respectively). The C20:1 content was not significantly different (*P* > 0.05) from those in rice PBMA, coconut PBMA, and skimmed milk (averaging 0.2% of TFAs). The other PBMA types and the milk groups had lower contents in the individual FAs mentioned above, with significant (*P* < 0.05) differences among them.

Furthermore, beyond the differences previously presented between milk groups and PBMA types, it is important to highlight that some variances within the samples of the same group were observed in PBMAs.

### 3.5 Principal Component Analysis

**Figure 1:**
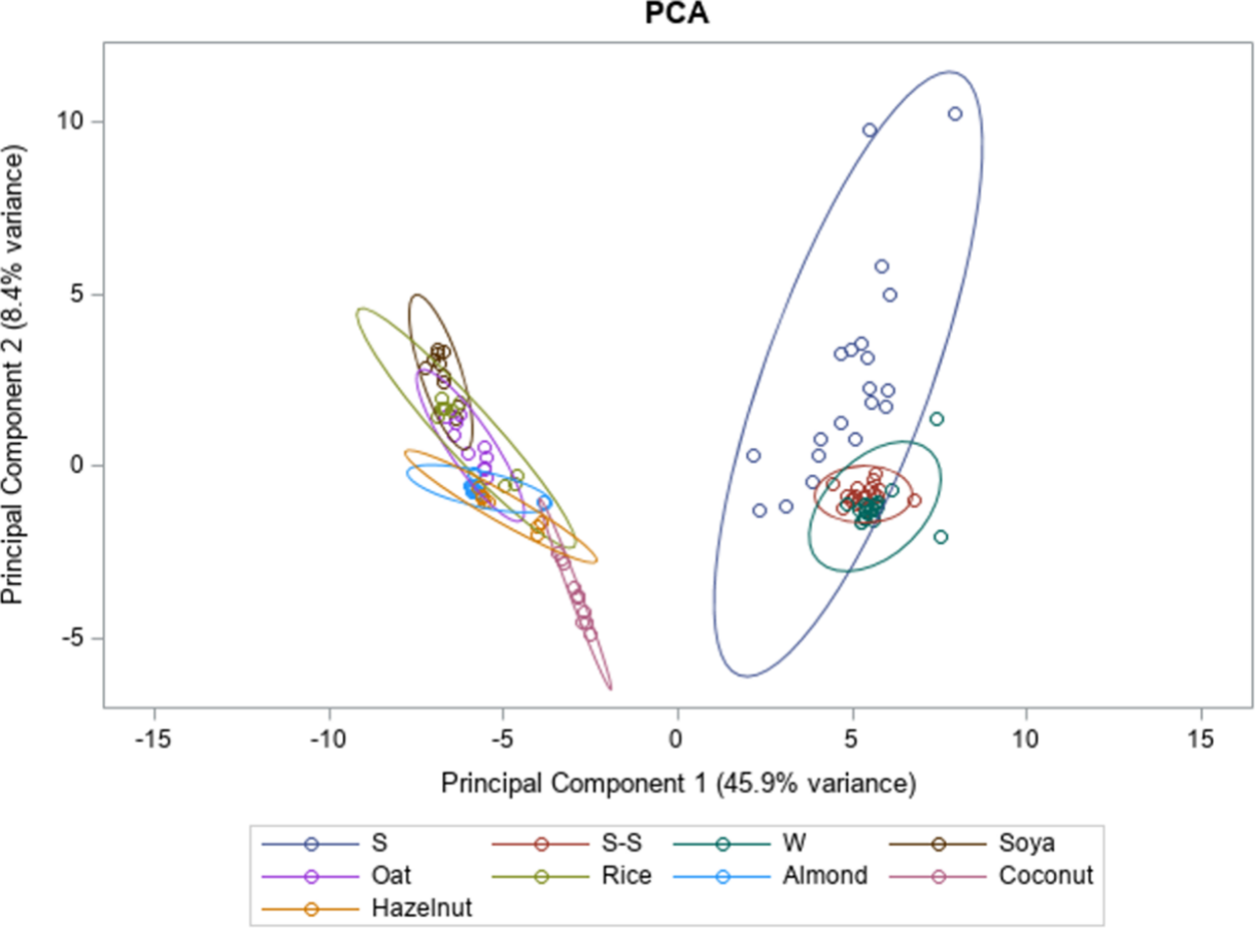
Principal component plot for the lipid profile of cow’s milk from different fat classes (S = skimmed milk; S–S = semi-skimmed milk; W = whole milk), and plant-based milk alternatives (soya, oat, rice, almond, coconut, and hazelnut).

A principal component analysis (PCA) was performed to describe the variability of the data in two dimensions. In Figure 1, the location of each beverage group in the multivariate space is depicted. The two principal components (PCs) explained 54.3% of the variability, 45.9% explained by PC1 and 8.4% explained by PC2. It is possible to clearly distinguish the milk samples from the PBMA samples, although coconut PBMA was the PBMA type closest to the milk groups. According to the size of their ellipses, in milk groups, skimmed milk and semi-skimmed samples showed the highest and lowest variability, respectively, while whole milk showed an intermediated variability. PBMA types exhibited variability both among different types and within the same type. This variability was most notable in rice PBMA, which had a larger ellipse. Additionally, there was a distinct separation between soya PBMA and hazelnut PBMA samples, whereas soya and coconut PBMAs showed the least variability between the samples.

## 4 Discussion

### 4.1 Total Lipids

The analysis of the total lipid (TL) content in both milk and plant-based milk alternative (PBMA) types reveals significant differences that might influence consumer choices based on dietary needs and preferences. In milk groups, variations are mainly due to the skimming process, underscoring the importance of checking nutritional labels and selecting products that match one’s dietary requirements. PBMA types exhibit a narrower range of TL contents, generally lower than that of whole milk but higher than that of skim milk. The relatively higher TL content in almond, coconut, and hazelnut PBMAs compared with soya, oat, and rice PBMAs could be attributed to the natural fat content of these raw materials. TL content is a critical factor for consumers concerned with their fat intake; therefore, skim milk and rice PBMA are the best options for those seeking to reduce dietary fat due to their lower lipid content.

### 4.2 Total Cholesterol

Cholesterol is a structural component of cellular membranes that plays a role in signaling processes and membrane fluidity and is a precursor of other steroid molecules, such as vitamin D, bile acids, and steroid hormones.^32,33^ This sterol can be synthesized by humans in the liver or can be obtained through the diet.^33,34^ Our findings on milk total cholesterol revealed much lower levels compared to those reported by others (10–14 mg/100 mL).^16,21^ The discrepancies may be linked to variations in lipid content, as cholesterol is associated with the lipid fraction of milk.^35^ Additionally, the significant reduction of total cholesterol in both semi-skimmed and skimmed milk is a positive attribute due to its negative implications on human health, namely, in the development of some chronic cardiovascular diseases.^6,7^

Cholesterol can be found in trace amounts in plant membranes;^1^ however, in the current work, this sterol could not be quantified in PBMAs. This may also be considered an advantage of these types of beverages over milk, since cholesterol, along with saturated fatty acids (SFA), is a considered risk factor for the development of some chronic cardiovascular diseases.^6,7^

### 4.3 Fatty Acid Partial Sums, Fatty Acid Ratios, and Lipid Quality Indices

The high milk total SFA content has been used to jeopardize its image among consumers.^11,18^ Conversely, PBMAs generally have better fatty acid (FA) profiles, with higher monounsaturated FA (MUFA) or polyunsaturated FA (PUFA) contents and lower SFA content (with the exception of coconut PBMA), which may be seen by consumers as an advantage, since these two groups of FAs are known for their antiatherogenic properties.^23^ However, the PUFA fraction of PBMAs is dominated by the n-6 PUFA family (averaging 97% of total PUFA) rather than the n-3 PUFA family (averaging 4% of total PUFA), contributing to the imbalance of the diet’s n-6/n-3 ratio, which is regarded as negative to human health.^2,36−39^ The n-6 PUFA and n-3 PUFA families use the same enzymes for elongation and unsaturation processes, and each family is a precursor of a specific type of eicosanoids,^2,40^ having the potential to produce different health outcomes.^2,38^ The very long chain n-3 PUFA, eicosapentaenoic acid (EPA) and docosahexaenoic acid (DHA), are known to reduce the risk of cardiovascular diseases due to their antiatherogenic, antithrombotic, and anti-inflammatory properties.^38,40−42^ This is a consequence of being precursors of eicosanoids with health benefits, namely, prostaglandin E3, thromboxane A3, prostacyclin I3, and leukotriene B5.^2,43^ On the other hand, eicosanoids synthesized from arachidonic acid (n-6 PUFA), namely, prostaglandin E2, thromboxane A2, prostacyclin I2, and leukotriene B4, have been associated with the increased risk of cardiovascular disease, inflammation, and cancer.^2,36,38,39,43^ Therefore, the higher PUFA of PBMAs, initially considered an advantage of PBMAs over milk, may also be seen as a risk to human health. Furthermore, the results obtained indicated that PBMAs presented more favorable values of P/S, due to their unsaturated fraction, and h/H than milk, indicating a predominance of FAs with hypocholesterolemic effects relative to FAs with hypercholesterolemic effects. Furthermore, the higher PI value of PBMAs results from their higher unsaturation degree and could be seen as a negative point regarding the stability and storage of the product, since these beverages are more prone to oxidation. Regarding the remaining lipid quality indices, milk presented worse AI and TI values, reflecting a greater atherogenic and thrombogenic potential than PBMAs.

### 4.4 Fatty Acid Profile

Milk is a complex matrix associated with more than 400 FAs.^35^ However, the FA profile presented herein encloses only 54 individual FAs (24 were SFA, 20 were MUFA, and 10 were PUFA). The SFA represent 70.6% of TFAs; however, individual SFAs have different biological activities, which may result in different health outcomes.^3,8^ The short-chain (C4:0 and C6:0) and medium-chain (C8:0 and C10:0) FAs, along with C18:0, were considered neutral regarding blood cholesterol.^3^ C4:0 has also been observed to have antitumor, antimicrobial, and anti-inflammatory activities and to be a promoter of gut health and integrity, while the intake of medium-chain FAs suppresses fat deposition in animal and human subjects.^8,44^ Furthermore, some studies indicate that odd-numbered straight-carbon-chain FAs, such as C15:0 and C17:0, could exert a beneficial effect on human health,^8^ and together, these FAs represent 14% of total SFA in milk. Moreover, milk has in its FA profile an emerging class of bioactive FAs, namely, methyl-branched-chain FAs (BCFA), which are known for their anticarcinogenic, anti-inflammatory, and energy and glucose homeostasis properties.^45^ The BCFA content presented herein for milk (1.5% of TFAs) is in the range of values shown by other studies.^45,46^

*Trans* FAs (*trans*FAs) are a group of unsaturated FAs (MUFA or PUFA) with *trans* configuration instead of *cis* configuration.^1^*Trans*FAs have two dietary sources, namely, (1) the natural source from ruminant-derived products, such as meat and dairy products and (2) the industrial source from hydrogenation of vegetable oils, deodorization of unsaturated vegetable oils high in PUFA, or heating/frying of oils at temperatures >220 °C.^1,47^ There is convincing evidence that *trans*FAs increase the risk of coronary diseases (CHD)^2^ and, in milk, they represent 2.7% of TFAs in both semi-skimmed and whole milk, which is a worrying percentage and can be seen as an advantage of PBMAs over milk, since in PBMAs, they are absent. However, in skimmed milk, the representation is lower (1.8% of TFAs), and some *meta*-analyses indicate that the consumption of milk (200 mL/day) is not associated with CHD, being inclusively responsible for a 7% lower risk of stroke.^48,49^ Furthermore, milk has rumenic acid (C18:2 *cis*-9,*trans*-11), the most representative isomer of conjugated linoleic acid (CLA),^1,50^ which has been associated with health-promoting properties in humans, namely, anticarcinogenic properties, atherosclerosis reduction, antioxidant properties, and immune stimulant.^51^ The majority of rumenic acid present in milk fat (70–90%) is synthesized through the endogenous conversion of vaccenic acid (C18:1 *trans*-11) into rumenic acid by the action of Δ9 desaturases.^1^ As stated before, these two FAs are health promoters.^52^

The term PBMAs encloses a series of beverages from different raw materials and formulations, leading to different products with nutritional quality dissimilarities.^18,53^ The lipid profile of the PBMA types analyzed demonstrated that there is considerable variation between beverages.

The higher content in n-6 PUFA shown by soya, oat, and rice PBMAs was mainly due to the C18:2 n-6 content, which is the main FA of their raw materials.^54−56^ Furthermore, C18:2 n-6 and C18:3 n-3 were prime FAs responsible for the results obtained in the n-6/n-3 ratio, and in this regard, soya PBMA presented a lower value due to the highest C18:3 n-3 content, while almond PBMA presented the highest value because of the lowest n-3 PUFA content. Finally, the higher contents in MUFA shown by almond and hazelnut PBMAs were the consequence of their higher contents of C18:1 *cis*-9, which is the most representative FA of their raw materials.^57^

Coconut PBMA was the beverage showing the worst FA profile with regard to human health. Its lipid profile is dominated by SFA, namely, C14:0 and C16:0, both with hypercholesterolemic effects.^2,3^ Its high total SFA content results in worse values of AI and TI, which means that this beverage has the greatest atherogenic and thrombogenic potential. Furthermore, results obtained in h/H ratios also indicate that in coconut PBMA, the contents of individual FAs with a hypercholesterolemic effect were higher than those with a hypocholesterolemic effect. On the other hand, a single positive attribute may be recognized in coconut PBMA, which is its lower susceptibility to oxidation, as indicated by the obtained PI value.

Lastly, the varying degrees of variance between samples of the same PBMA type can be attributed to differences in raw materials and the manufacturing process of these beverages.^16^ This variability complicates the establishment of a standard lipid profile for PBMA types.

### 4.5 Principal Component Analysis

In PCA, the observed variability between milk samples and PBMA samples may be attributed to specific differences in their lipid profiles. Milk has a more complex lipid profile, containing 54 individual FAs and total cholesterol, while PBMAs only include 15 FAs and lack cholesterol. Additionally, the unbalanced number of samples in each group might also contribute to the observed variability. The coconut PBMA was shown to be closer to the milk group than the remaining PBMA types, which could be a consequence of its SFA richness. Finally, the variability observed among PBMA types and among samples of the same PBMA type was already evident in variance analysis. Such a situation could be a consequence of differences in raw materials, as well as differences in beverage manufacturing processes.^16^

This PCA corroborated the earlier findings, revealing distinct lipid profiles between milk and PBMAs, as well as considerable variability among different PBMA types, with some groups showing more pronounced differences. Consequently, this variability hinders the establishment of a standard lipid profile for PBMA types and enabled us to reject the initially established scientific hypotheses, namely, (1) different PBMA types present similar lipid profiles and (2) the lipid profiles of PBMA and milk samples from different fat classes show no differences. Furthermore, this comparative analysis aids in understanding the composition differences and could guide consumers in making informed choices based on their dietary needs and preferences, particularly those related to fat intake, which can affect nutritional and health outcomes.

## Supporting Information Available

Additional information regarding the PCA performed, namely, the principal components’ eigenvalues, loadings of the variables, and the principal components’ loadings of the samples (PDF)
