# Supplementary material for: Lipid Profile of Plant-Based Milk Alternatives (PBMAs) and Cow’s Milk: A Comparison
Source: J Agric Food Chem. 2024 Aug 2;72(32):18110–20. doi: 10.1021/acs.jafc.4c03091 (PMC11328168; doi:10.1021/acs.jafc.4c03091)
Supplement: Supplementary file 1 — jf4c03091_si_001.pdf [file jf4c03091_si_001.pdf]

Lipid Profile of Plant-Based Milk Alternatives (PBMAs) and Cow's Milk: A Comparison

Irene Antunes,[†,‡] Ricardo Bexiga ,[†,‡] Carlos Pinto ,[§] Helena Gonçalves ,[‖] Cristina Roseiro ,[‖,⊥] Rui Bessa ,[†,‡] Susana Alves ,[‡] **and** Mário Quaresma,[†,‡,*]

[†] CIISA − Centre for Interdisciplinary Research in Animal Health, Faculty of Veterinary Medicine, University of Lisbon, 1300−477 Lisboa, Portugal

[‡] Associate Laboratory for Animal and Veterinary Sciences (AL4AnimalS), Faculty of Veterinary Medicine, University of Lisbon, 1300−477 Lisboa, Portugal

[§] Faculdade de Ciências Agrárias e do Ambiente, Universidade dos Açores, 9700−042 Angra do Heroísmo, Açores, Portugal

[‖] Food Technology and Safety Division, National Institute for Agricultural and Veterinary Research (INIAV, IP), Quinta do Marquês, 2780−159 Oeiras, Portugal

[⊥] GeoBioTec −Geobiosciences, Geoengineering e Geobiotechnologies, NOVA School of Science and Technology, Campus de Caparica, 2829−516 Caparica, Portugal

*Email: mquaresma@fmv.ulisboa.pt. Tel.: +351 213602042 .

Table 1 - Eigenvalues, % of explained variance, and cumulative variance (%).

Factors	Eigenvalue	Explained variance	Cumulative variance	Factors	Eigenvalue	Explained variance	Cumulative variance
1	30.5330266	0.4626	0.4626	34	0.0734969	0.0011	0.993
2	5.8909552	0.0893	0.5519	35	0.0669091	0.001	0.994
3	4.3513696	0.0659	0.6178	36	0.0456593	0.0007	0.9947
4	3.8040861	0.0576	0.6754	37	0.0417583	0.0006	0.9953
5	2.7988874	0.0424	0.7179	38	0.0394203	0.0006	0.9959
6	2.2811666	0.0346	0.7524	39	0.0377262	0.0006	0.9965
7	1.9664416	0.0298	0.7822	40	0.0337927	0.0005	0.997
8	1.7125718	0.0259	0.8082	41	0.0287232	0.0004	0.9974
9	1.5372589	0.0233	0.8315	42	0.0252834	0.0004	0.9978
10	1.0913584	0.0165	0.848	43	0.0231485	0.0004	0.9982
11	1.0115213	0.0153	0.8633	44	0.0183009	0.0003	0.9985
12	0.9571117	0.0145	0.8778	45	0.0160073	0.0002	0.9987
13	0.9455613	0.0143	0.8921	46	0.0148805	0.0002	0.9989
14	0.7754417	0.0117	0.9039	47	0.0138786	0.0002	0.9991
15	0.7061192	0.0107	0.9146	48	0.0113643	0.0002	0.9993
16	0.5744196	0.0087	0.9233	49	0.0104225	0.0002	0.9995
17	0.5505692	0.0083	0.9316	50	0.0077253	0.0001	0.9996
18	0.5006302	0.0076	0.9392	51	0.0058611	0.0001	0.9997
19	0.4465112	0.0068	0.946	52	0.0052197	0.0001	0.9998
20	0.4150404	0.0063	0.9523	53	0.00433	0.0001	0.9998
21	0.375978	0.0057	0.958	54	0.0029723	0	0.9999
22	0.3348629	0.0051	0.963	55	0.0024003	0	0.9999
23	0.2975727	0.0045	0.9676	56	0.0019693	0	0.9999
24	0.2709077	0.0041	0.9717	57	0.0014815	0	1
25	0.2295967	0.0035	0.9751	58	0.0008561	0	1
26	0.2068855	0.0031	0.9783	59	0.0007175	0	1
27	0.1721882	0.0026	0.9809	60	0.0006472	0	1
28	0.1607323	0.0024	0.9833	61	0.0004489	0	1
29	0.1431747	0.0022	0.9855	62	0.0002689	0	1
30	0.1238018	0.0019	0.9874	63	0.0001573	0	1
31	0.1194587	0.0018	0.9892	64	0.0000526	0	1
32	0.0979927	0.0015	0.9907	65	0.0000035	0	1
33	0.0809161	0.0012	0.9919	66	0.0000005	0	1

Table 2 - Principal component (PC) loadings of the variables (PC1-PC33).

Variable	PC1	PC2	PC3	PC4	PC5	PC6	PC7	PC8	PC9	PC10	PC11
TL	0.084245	-0.229	0.151983	0.016217	0.027213	0.085481	0.104793	0.169963	0.127621	-0.02724	-0.1646
C4:0	0.134398	0.032414	0.017633	-0.00899	-0.01112	-0.13521	-0.06691	-0.13218	0.032303	0.0612	-0.25396
C6:0	0.151153	0.006653	0.071713	-0.00706	0.021857	-0.00997	0.102085	0.004709	-0.05151	0.010196	-0.05859
C8:0	0.15034	-0.02748	0.102014	-0.00806	0.033296	0.096463	-0.10272	-0.04194	-0.00695	-0.04814	0.002954
C10:0	0.175293	-0.01726	0.08077	0.014418	0.018673	-0.00134	-0.00542	-0.03466	-0.02671	0.015645	-0.01641
C11:0[a]	0.162875	-0.02267	0.076951	0.015195	0.06372	0.059041	-0.09822	0.002441	-0.02271	0.009719	0.085817
C12:0	0.174414	-0.03113	0.0828	0.021125	0.02182	-0.01594	-0.04686	-0.02982	-0.00887	0.004346	0.020561
C13:0 *anteiso*	0.106726	0.104439	-0.01969	-0.03594	0.146467	-0.24852	-0.0082	-0.02087	-0.2069	0.040624	-0.25713
C12:1	0.131638	0.004274	0.021195	0.008789	-0.03712	-0.18916	-0.24388	-0.08684	0.075443	0.067881	-0.03127
C13:0	0.130663	0.000897	0.04065	0.002133	0.078415	-0.06079	-0.27877	-0.00957	-0.05114	-0.07731	0.072361
C14:0 *iso*	0.109946	0.136329	0.027413	-0.06109	-0.18752	-0.29751	0.109748	0.099438	0.109352	-0.04511	0.032017
C14:0	0.054529	-0.06049	-0.40414	0.168118	-0.01808	0.067377	0.055882	0.058133	0.031487	0.018039	0.063125
C15:0 *iso*	0.086153	0.154418	0.025432	-0.07539	-0.24391	0.280411	0.110253	-0.15809	0.156582	0.09372	-0.21164
C15:0 *anteiso*	0.153422	0.010178	0.088488	0.0045	-0.02992	-0.16941	0.111375	0.150221	0.079341	-0.04862	0.119486
C14:1 *cis*-9	0.169066	-0.05035	0.096131	0.024941	0.002235	0.090433	-0.05058	-0.04559	-0.04393	0.013132	0.026765
C15:0	0.163221	-0.03038	0.083971	0.021601	0.043766	0.074148	0.032501	-0.13437	0.068427	0.061865	0.032483
C16:0 *iso*	0.140192	0.02152	0.031383	0.006278	0.05495	-0.20896	0.073491	-0.20432	0.080818	0.100117	-0.29084
C16:0	0.174966	-0.01562	-0.03311	0.089658	0.014613	-0.00041	0.007357	-0.00386	-0.03188	0.001675	0.029562
C17:0 *iso*	0.127115	-0.01267	0.094804	-0.00267	-0.12975	0.019725	0.067417	0.361799	0.149989	0.041466	-0.14015
C16:1 *cis*-7	0.143426	0.038698	0.070951	-0.01521	0.135353	0.166915	-0.07422	0.003307	0.045075	-0.01345	0.084029
C16:1 *cis*-9	0.146601	-0.09707	0.112466	-0.00738	-0.01746	0.076701	0.083577	-0.12122	0.016262	0.042753	0.13924
C17:0	0.122609	-0.06849	0.126986	0.033577	-0.01768	0.112079	0.163323	-0.1079	0.093704	-0.06318	0.176846
C17:1 *cis*-9	0.142916	0.007802	0.108396	0.006118	-0.09529	0.053105	0.191897	-0.01378	0.147609	-0.09662	0.066999
C18:0	0.16407	0.066568	-0.08082	0.079581	0.001865	-0.08884	-0.05673	-0.028	-0.05379	0.017028	-0.02032
C18:1 *trans*-6[a]	0.146013	-0.07795	0.122173	0.037473	0.004839	0.164285	-0.08691	-0.00968	0.059928	-0.02179	-0.00115
C18:1 *trans*-9	0.128894	-0.01906	0.069461	0.008406	-0.06677	-0.01335	-0.38349	-0.0332	0.082451	-0.04941	0.082062
C18:1 *trans*-10	0.146576	-0.1202	0.124822	0.060634	0.024655	0.046635	-0.02025	-0.05091	-0.01525	-0.02058	0.012476
C18:1 *trans-11*	0.14991	-0.10048	0.124082	0.048347	0.028417	0.068918	0.011088	-0.01691	-0.01749	-0.01448	0.092176
C18:1 *trans*-12	0.149469	-0.10526	0.126646	0.05186	-0.03544	0.074472	0.014521	0.025591	0.035899	-0.05478	-0.13062
C18:1 *cis*-9	-0.10681	-0.09345	0.049315	-0.3774	0.01063	-0.02513	0.007179	-0.01519	0.004151	0.099889	0.047219
C18:1 *trans*-15	0.137312	-0.08079	0.109932	0.0529	0.04982	-0.06063	0.116989	-0.09451	-0.06382	-0.09897	0.150833
C18:1 *cis*-11	-0.11205	-0.06313	0.262925	-0.08009	0.052602	0.031337	0.081452	0.012846	-0.00716	0.149444	0.17993
C18:1 *cis*-12	0.07376	0.141846	0.009184	-0.06166	0.433936	0.124595	0.055287	0.134446	0.091132	-0.13293	0.084676
C18:1 *cis*-13	0.051663	0.189624	-0.03061	-0.08845	0.417836	0.128695	-0.02011	0.159256	0.086841	-0.07295	0.096799
C18:1 *trans-16*[b]	0.073954	0.008423	0.046765	-0.02812	-0.15297	-0.02488	-0.07005	0.611304	0.050665	0.048686	-0.07402
cyclo-17[d]	0.145445	0.001717	0.076274	0.000562	-0.0425	-0.0786	0.135586	0.008766	-0.23714	-0.04275	-0.07887
C18:1 *cis-16*	0.084624	0.053555	0.001511	-0.04103	-0.01914	0.109313	-0.08876	0.079674	-0.6379	-0.01562	-0.08263
C18:2 *trans*-9,*cis-12*	0.096677	0.149223	0.001508	-0.06632	-0.03115	0.034194	-0.2977	-0.14909	-0.05868	-0.02545	0.078455
C18:2 *trans*-11,*cis*-15	0.095664	0.079301	-0.00714	-0.03592	-0.08306	-0.26761	-0.39059	-0.02057	0.130579	0.004958	0.084251
C18:2 n-6	-0.14686	0.100246	0.173647	0.156013	-0.02122	-0.00073	-0.03395	-0.00677	0.002728	-0.16198	-0.07913
C19:1	0.120791	0.112339	0.025318	-0.03887	-0.04054	-0.30715	0.230291	0.000921	0.078946	-0.03279	0.016107
C20:0	0.108843	0.188672	-0.00926	-0.08517	-0.04681	-0.04982	0.000407	-0.01205	0.189719	0.138553	-0.08413
C18:3 n-6	0.077641	0.225135	-0.02791	-0.13278	-0.21696	0.085388	-0.18591	0.268049	-0.04897	0.08148	0.069641
C20:1	-0.02519	0.306826	-0.019	0.07074	0.280813	0.02084	0.07034	0.045981	0.048861	0.07431	-0.01268
C18:3 n-3	-0.0724	0.11545	0.170262	0.290297	0.028452	-0.0019	-0.00014	0.031857	-0.04865	0.479607	0.117278
C18:2 *cis*-9,*trans*-11	0.146854	-0.0059	0.088869	0.00786	0.087063	0.001903	0.065728	-0.11709	0.020881	-0.0256	-0.03478
C21:0	0.054583	0.230031	0.00867	-0.09978	-0.27596	0.220815	-0.00738	-0.09167	0.088341	0.110266	0.187627
C20:2 n-6	0.058052	0.259813	-0.0368	-0.09811	0.310253	0.080567	-0.09024	0.062623	0.175584	0.003223	-0.06422
C22:0	-0.08331	0.213683	0.116959	0.180693	-0.13904	-0.04023	-0.00088	-0.00743	0.075146	-0.40409	0.100543
C20:3 n-6	0.110415	0.199834	0.014372	-0.0967	-0.15042	0.164566	0.126777	-0.08275	-0.11301	0.029697	0.003568
C20:4 n-6	0.085676	0.272997	-0.0412	-0.12396	0.074266	-0.04667	0.192852	-0.10409	-0.05591	0.048334	-0.25502
C23:0	0.047215	0.196995	-0.01057	-0.06641	-0.05734	-0.29028	0.210862	-0.00682	-0.02761	-0.0562	0.447417
C24:0	0.005654	0.263424	-0.10727	-0.06327	-0.20397	0.305341	0.017056	-0.08626	0.012692	-0.0681	-0.08999
SFA	0.16306	-0.01962	-0.17085	0.117764	-0.00361	0.007604	0.014208	0.003004	-0.00081	0.016065	0.019004
MUFA	-0.07459	-0.1101	0.071402	-0.40586	0.037939	-0.01361	0.013982	0.003529	0.004368	0.190575	0.067755
PUFA	-0.14272	0.116035	0.184071	0.172286	-0.01651	-0.00205	-0.02996	-0.0049	-0.00548	-0.11241	-0.06961
n-6 PUFA	-0.14843	0.107745	0.157608	0.161556	-0.02056	0.003746	-0.03002	-0.00451	0.00297	-0.16061	-0.08343
n-3 PUFA	-0.06764	0.118637	0.164145	0.286844	0.031563	-0.01592	0.006791	0.029147	-0.04715	0.510991	0.103023
AI	0.069147	-0.07003	-0.37313	0.180083	-0.02084	0.056022	0.064548	0.040686	0.056364	0.019491	0.073018
TI	0.115854	-0.05934	-0.28972	0.15978	-0.01856	0.028967	0.045055	0.020799	0.046477	0.01321	0.06231
P/S	-0.15436	0.08099	0.174625	0.10297	-0.0235	-0.0012	-0.02332	-0.00268	0.012207	-0.13943	-0.0638
n-6/n-3	-0.08431	-0.06737	0.027173	-0.2715	-0.01318	-0.01286	-0.03289	-0.03221	0.042945	-0.10094	-0.02012
PI	-0.14457	0.125929	0.16827	0.183755	-0.01054	-0.00028	-0.01675	-0.00425	-0.00722	-0.0573	-0.06697
h/H	-0.15484	-0.02619	0.117405	-0.19331	-0.00161	-0.01594	0.007315	-0.00834	0.02372	0.033617	0.019479
C18:2 *cis,trans/trans,cis*[c]	0.108486	0.116931	0.034247	-0.0712	-0.02646	0.00765	0.109672	0.101284	-0.41331	-0.10959	0.156587
CHL	0.122076	-0.08309	0.112179	0.021231	-0.01054	0.056615	0.060643	0.24428	-0.03145	-0.0176	-0.16153

Variable	PC12	PC13	PC14	PC15	PC16	PC17	PC18	PC19	PC20	PC21	PC22
TL	0.193912	0.090043	-0.16889	-0.0566	-0.21431	0.073741	0.073477	0.083449	0.225798	0.102496	0.249135
C4:0	-0.00859	-0.30347	-0.13304	-0.33581	0.260611	-0.06993	0.094866	0.09193	-0.2067	0.06643	0.009669
C6:0	-0.13067	-0.07695	-0.04438	-0.02851	-0.22088	-0.1639	0.166786	-0.14576	0.079343	0.272705	0.217933
C8:0	-0.02222	0.0931	-0.08564	-0.35617	-0.09367	0.023832	-0.05385	-0.22167	0.000176	0.294764	-0.11594
C10:0	-0.08369	-0.07706	-0.00504	0.02319	-0.0056	0.011635	0.008514	0.020611	-0.03249	-0.00612	0.075181
C11:0[a]	-0.10657	-0.19884	0.11771	-0.03616	0.035884	0.056225	-0.03557	-0.01004	0.005779	-0.09862	0.103997
C12:0	-0.07303	-0.08739	0.019433	-0.00471	-0.01787	0.022375	-0.00276	0.030493	-0.06285	-0.03087	0.056113
C13:0 *anteiso*	-0.11411	0.281343	0.225716	0.183003	-0.16158	-0.03489	0.045024	0.189411	-0.16776	0.113624	-0.00485
C12:1	0.031409	-0.27095	0.144544	0.305308	-0.23659	-0.0331	-0.12494	-0.08705	0.01913	0.060212	-0.16307
C13:0	0.21198	-0.35048	-0.02582	0.099426	-0.02256	-0.15523	0.121279	0.232572	0.208359	0.148168	-0.05114
C14:0 *iso*	0.283688	0.056706	-0.01822	0.07256	-0.09959	0.073722	-0.06325	-0.14011	0.064356	-0.0487	0.071746
C14:0	0.095387	0.089168	-0.01333	-0.03301	0.006352	0.036042	0.018181	-0.04728	0.067797	0.038363	-0.08936
C15:0 *iso*	0.164352	-0.08523	0.176304	-0.04068	0.015125	-0.06942	0.025805	0.055933	-0.04805	0.100381	-0.08779
C15:0 *anteiso*	0.021247	0.038377	0.106099	0.086362	0.110216	-0.0427	-0.05115	-0.23677	0.01445	0.020221	-0.05859
C14:1 *cis*-9	-0.07068	-0.06257	0.003786	0.100464	-0.05668	-0.03329	-0.00213	-0.07557	-0.06756	0.020451	0.075251
C15:0	-0.1959	-0.17112	-0.0273	0.095488	0.013872	0.011388	-0.072	0.003426	0.008227	0.029775	-0.01727
C16:0 *iso*	-0.07728	0.005566	0.048078	-0.0187	0.062765	0.139128	-0.107	-0.15103	0.144557	-0.20558	0.124497
C16:0	-0.06748	-0.05867	0.046879	0.056689	-0.06192	0.009917	0.029328	-0.02053	-0.01966	-0.00651	0.021749
C17:0 *iso*	-0.07813	0.056972	0.222566	0.064687	0.134615	-0.08036	0.051685	0.086178	-0.03778	0.047728	0.090034
C16:1 *cis*-7	-0.08872	0.115955	-0.29882	0.21985	0.142213	-0.13698	0.07659	-0.1355	-0.08453	0.09705	-0.02175
C16:1 *cis*-9	-0.24861	-0.139	0.080998	0.136133	-0.11125	0.099871	0.010133	0.011687	0.186557	-0.02227	0.080917
C17:0	0.088373	-0.00547	0.081053	-0.1405	-0.13398	-0.18429	0.297731	-0.19738	-0.21234	-0.56426	0.124755
C17:1 *cis*-9	0.296319	-0.05997	0.004388	-0.03039	0.012229	-0.00903	-0.02935	-0.05534	-0.26788	0.171438	-0.0483
C18:0	0.043562	0.062736	-0.02113	-0.0376	0.01161	0.037082	0.057106	-0.06934	-0.13896	-0.03945	-0.01975
C18:1 *trans*-6[a]	0.063092	0.144543	-0.1425	0.141912	0.23259	-0.09952	0.117068	0.230126	0.100969	-0.1377	-0.02829
C18:1 *trans*-9	0.225657	0.183048	-0.15871	0.082099	-0.08133	0.083524	-0.01145	0.081849	0.078244	0.007129	0.112258
C18:1 *trans*-10	-0.06592	0.099293	0.057538	-0.02443	0.376679	-0.09172	-0.01001	-0.00277	0.117616	0.082782	-0.1438
C18:1 *trans-11*	-0.16736	0.20671	0.055998	-0.05351	0.223729	0.073642	-0.10114	0.009011	0.01986	-0.08886	-0.09732
C18:1 *trans*-12	0.196765	0.108745	-0.0677	-0.03942	-0.06533	0.03484	0.07927	0.23435	-0.1926	-0.00627	0.066586
C18:1 *cis*-9	0.055631	0.052833	0.000722	-0.0307	-2.9E-05	-0.103	-0.06671	0.054985	0.020358	-0.01187	-0.03438
C18:1 *trans*-15	0.162985	0.03829	0.173313	0.175627	0.05203	0.087016	-0.18223	0.156428	-0.10446	-0.12906	-0.37708
C18:1 *cis*-11	0.181568	-0.06634	0.102897	0.194842	0.186353	0.046582	-0.12289	0.005106	0.103775	0.025526	0.126622
C18:1 *cis*-12	0.058791	-0.0393	0.118971	-0.17221	-0.01778	0.095768	-0.04508	0.000216	-0.02871	0.089283	0.043079
C18:1 *cis*-13	-0.0011	-0.12069	-0.01923	-0.04428	-0.09245	0.092498	-0.07805	-0.04247	-0.13345	0.041219	-0.07328
C18:1 *trans-16*[b]	-0.15867	-0.06479	0.17158	-0.04348	0.170242	-0.04905	-0.00354	-0.00179	-0.07374	0.053641	-0.04934
cyclo-17[d]	0.101596	0.09365	-0.06258	0.079238	-0.11537	0.073739	0.006615	0.23165	-0.26693	0.017923	-0.00685
C18:1 *cis-16*	0.027371	-0.03889	0.021677	-0.03138	0.057026	0.016446	0.09348	0.077287	-0.03161	-0.11047	0.16174
C18:2 *trans*-9,*cis-12*	0.069774	0.321036	0.328843	-0.01156	0.005704	-0.28037	0.238144	-0.222	0.190065	0.085978	0.035275
C18:2 *trans*-11,*cis*-15	0.045961	0.079535	-0.01789	-0.3115	0.089929	0.193656	-0.11643	0.064406	-0.0732	-0.14042	-0.07434
C18:2 n-6	-0.06585	-0.03741	-0.00319	0.049945	0.03135	0.054413	-0.00085	0.004672	0.010437	-0.02491	0.03929
C19:1	0.035208	-0.1209	-0.14752	-0.06688	0.169702	-0.08236	0.132303	0.132111	0.245773	0.043448	0.005427
C20:0	-0.19152	0.137616	-0.43416	0.279418	0.030277	-0.02706	-0.00275	-0.1079	-0.14532	0.010121	-0.05356
C18:3 n-6	-0.02992	-0.03315	-0.09626	0.05041	-0.02415	0.112385	-0.06257	-0.11637	0.020093	-0.25393	0.20352
C20:1	0.056929	0.183556	-0.07982	0.169924	0.022788	-0.07282	0.037289	0.038763	-0.06421	-0.01287	-0.01202
C18:3 n-3	0.114968	0.010684	0.003226	-0.09476	-0.02724	0.0569	0.126301	0.002829	0.014128	0.061726	-0.06947
C18:2 *cis*-9,*trans*-11	-0.09952	0.265962	-0.02459	-0.16607	0.009595	0.299496	-0.16522	0.101386	0.241584	0.051092	0.167142
C21:0	-0.14852	-0.0265	0.015958	-0.0023	-0.19598	0.234394	-0.10116	0.232205	-0.09967	0.058134	0.060991
C20:2 n-6	0.084695	-0.07477	0.132657	-0.01253	0.004453	0.02038	0.053461	0.195514	0.105766	-0.19755	0.067453
C22:0	0.062014	0.055555	0.065212	-0.00457	-0.06093	-0.06039	-0.00602	-0.01472	0.019163	0.125357	0.076774
C20:3 n-6	0.031903	0.064151	0.21996	-0.0197	0.065218	0.129542	-0.16406	-0.08272	0.029	0.189985	0.016239
C20:4 n-6	0.147299	-0.05399	0.041302	0.0279	0.095838	0.001112	-0.00514	-0.1265	0.195731	-0.13626	-0.00137
C23:0	-0.27053	0.05447	-0.0241	-0.14893	-0.06246	-0.11773	0.218355	0.294003	0.058603	0.041812	-0.0098
C24:0	-0.03613	-0.02074	-0.09378	-0.04225	0.041157	-0.12535	0.044761	0.166449	0.17801	-0.06791	-0.2471
SFA	0.002774	-0.01215	0.008394	-0.00732	-0.00898	0.021972	0.031292	-0.02939	-0.01611	0.00915	-0.0149
MUFA	0.084904	0.06449	-0.00777	-0.02053	-0.01555	-0.07861	-0.01832	0.055345	0.007206	0.030882	0.02289
PUFA	-0.04969	-0.03429	-0.00403	0.038748	0.031101	0.06417	0.006453	0.001895	0.019907	-0.02278	0.036101
n-6 PUFA	-0.06039	-0.03172	-0.00495	0.045862	0.030598	0.050068	-0.00211	-0.00585	0.01033	-0.02705	0.015184
n-3 PUFA	0.12391	0.015618	-0.00155	-0.08382	-0.02296	0.069433	0.137894	-0.0163	0.032829	0.049286	-0.07122
AI	0.085078	0.000768	0.042614	0.093924	0.093551	0.099554	0.030499	0.073298	0.105878	0.029226	0.110937
TI	0.051499	-0.04819	0.058128	0.129065	0.099592	0.100626	0.042404	0.107412	0.075029	0.01383	0.182852
P/S	-0.05398	-0.02851	-0.00579	0.036917	0.030891	0.044139	0.015675	0.031434	0.032894	-0.01287	0.037484
n-6/n-3	-0.00954	-0.03821	0.067618	0.149099	0.12201	0.587081	0.649342	-0.12764	-0.03715	0.108092	-0.16723
PI	-0.02673	-0.02659	-0.00166	0.026749	0.027257	0.054829	0.017557	-0.01091	0.024453	-0.01909	0.003144
h/H	0.027154	0.013831	0.003546	0.009759	0.0261	-0.09395	-0.08798	0.112949	0.042646	-0.01339	0.107776
C18:2 *cis,trans/trans,cis*[c]	0.185264	-0.12201	-0.28567	0.003779	0.110599	0.095951	-0.11471	-0.20028	0.112584	0.015803	-0.06717
CHL	-0.07381	0.039771	-0.05928	-0.08729	-0.37454	0.024859	0.047515	0.013222	0.319409	-0.20125	-0.49544

Variable	PC23	PC24	PC25	PC26	PC27	PC28	PC29	PC30	PC31	PC32	PC33
TL	0.291864	-0.09565	0.04672	0.047782	0.306597	-0.19329	-0.193	0.244463	-0.17731	-0.00587	-0.34491
C4:0	0.200776	0.181206	-0.11652	0.049591	-0.06834	0.084943	-0.03268	0.017122	0.140885	-0.02449	0.002093
C6:0	0.20334	-0.20899	-0.2936	-0.42487	0.007957	0.062744	0.34632	0.069366	0.259626	0.064961	0.168228
C8:0	-0.20684	0.081224	0.212771	-0.02404	0.11302	0.041112	0.004098	-0.05085	0.01268	-0.0694	0.022559
C10:0	0.002852	0.010923	0.015691	0.080323	-0.03171	-0.01968	0.005406	-0.08139	-0.10405	-0.04407	0.048944
C11:0[a]	0.076809	0.123423	0.206546	-0.08988	0.110733	-0.1101	-0.0496	0.117201	0.165698	-0.27222	0.048187
C12:0	0.018604	-0.00043	0.032516	0.049397	-0.02039	-0.02659	-0.01762	-0.03814	-0.07749	-0.02658	0.05287
C13:0 *anteiso*	0.067582	-0.1787	-0.22732	0.115136	0.026085	0.112986	-0.15809	0.002329	-0.22633	-0.14332	0.126715
C12:1	-0.01048	0.01478	0.042414	0.15936	-0.05271	-0.1148	-0.12684	0.182502	0.067356	0.302677	0.003261
C13:0	-0.1982	-0.00613	-0.01797	0.03151	0.202792	0.171679	-0.01636	-0.10187	0.035211	0.118998	-0.0346
C14:0 *iso*	-0.15337	-0.11211	0.050967	-0.07889	-0.13259	-0.14247	0.187307	-0.03585	0.046355	-0.03107	0.019901
C14:0	-0.06149	0.051206	-0.02482	-0.10344	0.038679	0.028655	-0.04343	0.170862	0.021622	-0.0298	0.096219
C15:0 *iso*	-0.02272	0.101837	0.181143	-0.05146	0.014075	0.043553	0.171147	0.006102	-0.24209	0.181459	-0.05562
C15:0 *anteiso*	0.089353	-0.18435	0.146517	-0.00279	-0.13328	-0.09462	-0.15625	0.101056	-0.06505	0.014666	0.051392
C14:1 *cis*-9	0.006075	0.000643	0.01543	-0.00252	-0.06893	0.087279	-0.0483	0.141485	0.051655	0.016911	-0.03302
C15:0	0.017838	0.004409	-0.03319	-0.0621	0.035768	0.008164	-0.07271	0.122964	-0.02327	-0.00602	0.082328
C16:0 *iso*	0.032769	-0.04311	0.036669	0.150718	0.011848	-0.00999	0.194744	-0.18551	0.006254	-0.0201	-0.04964
C16:0	-0.01002	-0.04257	-0.03251	-0.0689	-0.01477	-0.0578	-0.06279	-0.13619	-0.0549	-0.07517	-0.04715
C17:0 *iso*	-0.28055	0.215131	-0.09711	0.12214	0.090776	-0.07872	0.021225	-0.03944	0.145597	-0.16089	0.033717
C16:1 *cis*-7	0.004266	-0.00705	0.006944	0.282022	-0.08558	0.11281	0.065543	-0.07835	-0.10379	-0.06123	-0.12813
C16:1 *cis*-9	-0.13503	0.013383	0.163462	0.012548	0.008789	-0.13776	0.011194	-0.07555	-0.05003	-0.05681	0.140964
C17:0	-0.01292	-0.0878	-0.06094	0.097086	0.103055	0.160893	-0.22683	0.007723	-0.01479	0.097248	0.141303
C17:1 *cis*-9	0.123802	0.117084	-0.13325	0.136955	-0.009	-0.24789	0.008693	-0.09697	0.123651	-0.02163	0.03113
C18:0	-0.00268	0.057329	-0.17014	0.027046	0.01826	0.058718	0.045624	-0.0883	-0.09117	0.467748	-0.13068
C18:1 *trans*-6[a]	-0.08323	-0.03483	0.007333	-0.14417	-0.05051	0.1082	0.217786	0.061542	-0.03803	0.023857	-0.06248
C18:1 *trans*-9	-0.06042	-0.14276	0.094522	-0.03729	-0.04775	0.075274	0.000466	-0.1776	-0.04284	-0.02524	0.184191
C18:1 *trans*-10	0.124279	-0.18	-0.03539	-0.11974	-0.17444	0.107528	-0.10703	0.159616	-0.24057	-0.04325	0.135491
C18:1 *trans-11*	-0.09595	0.023785	0.004189	-0.02613	0.008211	-0.09027	0.148348	0.269862	0.141279	0.284744	-0.12583
C18:1 *trans*-12	-0.05743	-0.04292	0.105779	-0.05197	-0.11854	-0.10639	0.132345	-0.19986	-0.10198	-0.03878	0.243806
C18:1 *cis*-9	0.029447	-0.01093	-0.01419	0.101805	-0.01754	0.064598	0.110699	0.068741	0.070029	-0.02942	-0.11302
C18:1 *trans*-15	0.136577	-0.05558	-0.17105	-0.06705	0.382923	-0.05106	0.273564	0.01734	0.069561	-0.09557	-0.08277
C18:1 *cis*-11	0.239265	0.15489	-0.17513	-0.18555	-0.26362	0.060217	-0.20029	-0.33171	0.08313	0.012983	-0.15473
C18:1 *cis*-12	-0.03125	-0.10544	-0.03603	0.114648	-0.1582	0.007335	0.018422	-0.07758	0.021633	0.01161	0.035195
C18:1 *cis*-13	0.141853	-0.18084	0.160024	0.046744	-0.14173	-0.02728	0.127975	0.026477	-0.06413	0.09782	-0.14156
C18:1 *trans-16*[b]	0.006923	-0.11171	0.018372	0.025735	0.050801	-0.01419	-0.00239	-0.05274	-0.04046	0.162319	0.078114
cyclo-17[d]	-0.12213	-0.03297	0.294658	-0.07004	-0.29247	0.209556	-0.17052	0.233422	0.427335	-0.0708	-0.18399
C18:1 *cis-16*	0.14787	0.099754	0.10336	0.080978	0.035879	-0.29866	0.066673	-0.01229	-0.10216	0.143258	-0.05054
C18:2 *trans*-9,*cis-12*	0.118537	0.201532	0.015392	0.057368	-0.11721	-0.19761	0.010343	0.015928	0.135334	-0.14432	-0.13543
C18:2 *trans*-11,*cis*-15	0.268996	-0.03987	0.045655	-0.15046	0.044993	-0.06133	-0.05854	0.055482	-0.02789	-0.08544	0.068057
C18:2 n-6	0.048391	-0.005	0.062157	-0.01603	-0.00537	-0.01346	0.019133	-0.0522	0.024104	0.045654	0.037114
C19:1	-0.08679	0.026557	0.017176	-0.05542	-0.03979	0.015457	-0.25719	0.075636	-0.13341	0.015591	-0.07879
C20:0	0.149048	0.109686	0.027084	0.119478	0.197506	-0.04879	0.005523	-0.03335	0.119962	-0.10357	-0.00738
C18:3 n-6	0.007652	-0.05624	-0.09334	-0.10537	0.046683	0.250189	0.044785	0.039546	0.053564	0.045795	-0.15107
C20:1	0.044097	0.320361	0.160558	-0.37934	0.168212	-0.13142	-0.20866	-0.00465	0.007098	0.223635	0.227012
C18:3 n-3	-0.02685	-0.09803	-0.025	0.137934	0.007242	0.028467	0.053987	0.079532	0.025499	-0.05231	-0.0033
C18:2 *cis*-9,*trans*-11	-0.16379	0.083867	-0.22778	0.172644	-0.00868	-0.01642	-0.12676	-0.0522	0.217967	0.287057	0.062467
C21:0	0.01344	0.130954	-0.21149	-0.07459	-0.22716	-0.08811	-0.01308	0.181265	-0.27922	-0.07682	-0.0024
C20:2 n-6	-0.11898	0.068187	-0.18052	-0.00235	0.10514	0.021811	-8E-06	0.130551	0.067304	-0.21329	-0.01195
C22:0	0.090853	0.168637	-0.14113	0.200396	-0.0096	0.235407	0.038182	0.253729	-0.06397	0.036615	0.026367
C20:3 n-6	0.117651	-0.11575	0.202788	-0.01308	0.356804	0.409963	-0.17893	-0.20252	0.053682	-0.03192	-0.01174
C20:4 n-6	-0.06608	-0.02798	0.133958	-0.04456	-0.11898	0.033847	0.160227	0.158974	-0.12836	-0.03993	-0.00618
C23:0	0.063887	0.012962	0.192219	0.052349	0.072368	-0.02534	0.162215	-0.171	-0.03472	0.05634	-0.11656
C24:0	0.067662	-0.45254	-0.10238	0.112769	-0.04759	-0.29502	-0.23123	-0.10783	0.308064	0.042019	0.05847
SFA	-0.01877	0.0227	-0.04142	-0.04961	0.001751	-0.00764	-0.03998	-0.00117	-0.02689	0.00435	0.017528
MUFA	0.05791	0.036792	0.065002	0.061357	0.060881	0.004667	-0.03572	0.107412	0.041288	0.229052	0.399944
PUFA	0.042898	-0.01317	0.057138	0.000604	-0.00404	-0.00736	0.025732	-0.04033	0.027371	0.039554	0.039707
n-6 PUFA	0.016466	-0.00469	0.05024	-0.06371	0.013996	-0.03556	-0.01516	-0.05705	0.011246	0.048904	0.002359
n-3 PUFA	-0.03228	-0.09188	-0.01187	0.124124	0.011797	0.007426	0.045678	0.073295	0.030032	-0.02462	0.021323
AI	0.198947	0.046847	0.109979	0.135981	-0.05997	0.079405	0.080357	0.045377	0.088867	0.006739	0.143726
TI	0.268073	0.039103	0.10811	0.191107	-0.07496	0.082557	0.092126	-0.05944	0.094072	0.044475	0.10273
P/S	0.069732	-0.05328	0.098137	0.113976	0.040575	0.004117	0.082899	0.163739	0.07449	-0.04378	0.232601
n-6/n-3	-0.00646	-0.00638	-0.02457	-0.01736	0.029506	0.012426	-0.00968	0.044273	0.033452	0.021166	0.015106
PI	0.009653	-0.02431	0.050073	-0.02702	0.008935	-0.02334	0.007674	-0.02669	0.010619	0.027388	-0.00053
h/H	0.138183	0.016852	0.117999	0.217117	0.018688	0.083833	0.144878	0.166268	0.03657	0.036787	0.20618
C18:2 *cis,trans/trans,cis*[c]	-0.09979	0.103865	-0.20125	0.113904	0.056436	-0.05392	-0.01006	0.081988	-0.08896	-0.15455	0.248143
CHL	0.259388	0.315055	0.007907	0.030448	-0.20416	0.210078	0.021656	-0.13637	0.02069	-0.02764	0.080123

[a]C18:1 *trans*-6/*trans*-7/*trans*-8 (co-elution); [b]C18:1 *trans*-16/*cis*-14 (co-elution); [c]Sums of several isomers with unknown geometry; [d]11-Cyclohexyl-11:0; TL= Total lipids; CHL= Total cholesterol.

Table 3 - Principal component (PC) loadings of the variables (PC34-PC66).

Variable	PC34	PC35	PC36	PC37	PC38	PC39	PC40	PC41	PC42	PC43	PC44	PC45	PC46	PC47	PC48	PC49	PC50
TL	0.16233	-0.07015	0.078059	-0.00701	-0.00029	0.091355	-0.02621	-0.11619	0.081829	-0.01421	-0.0498	-0.00832	0.002895	0.033966	0.003316	0.011978	0.062559
C4:0	0.073994	-0.14431	0.15135	-0.02781	0.067871	0.288061	0.103034	-0.1673	-0.14502	-0.16711	0.084646	0.022326	0.088185	0.188106	0.06129	0.147635	0.13285
C6:0	-0.04954	0.08791	-0.03	-0.05179	-0.08258	-0.14187	0.077854	-0.04638	-0.01208	0.012787	-0.06707	0.093088	0.017068	-0.06953	-0.02839	-0.11668	0.003562
C8:0	0.105852	0.137633	0.018807	0.064465	0.080231	0.085895	0.243244	0.164773	0.228191	0.334532	0.136437	0.070338	0.18638	0.023504	0.041681	0.08498	0.012227
C10:0	0.06386	-0.09938	-0.06862	0.07477	-0.12497	0.140237	-0.03028	0.115314	0.135596	-0.02058	0.248036	0.059563	-0.0716	-0.04148	-0.09966	-0.16982	-0.20567
C11:0[a]	-0.07281	-0.10681	-0.24016	-0.01191	-0.02001	0.019032	-0.03711	-0.19586	0.123452	0.128329	0.193095	0.025799	-0.25187	-0.26308	0.075583	0.071412	-0.02682
C12:0	0.048843	-0.14071	-0.09357	0.030632	-0.14107	0.090151	0.034374	0.143735	0.130181	-0.04833	0.217012	0.095149	-0.09584	-0.01717	-0.04949	-0.12701	-0.25635
C13:0 *anteiso*	0.127163	-0.0633	0.091699	-0.12271	0.005251	0.007761	0.085326	0.203705	0.117376	0.106882	0.070354	-0.07079	-0.14115	0.081234	0.100021	0.031069	0.048903
C12:1	-0.10727	0.064737	-0.07599	-0.06614	-0.04129	0.207783	0.222091	-0.13721	-0.01777	0.017145	-0.18211	0.09542	-0.08629	-0.01425	0.054036	-0.12332	0.179477
C13:0	0.138361	0.026833	0.310023	-0.06233	0.029666	-0.15412	-0.237	0.151127	-0.13157	0.133689	0.111849	-0.06317	0.059844	-0.11254	-0.08973	0.085172	-0.01887
C14:0 *iso*	0.022805	-0.19385	0.050441	0.008424	0.201781	0.065944	0.222153	-0.01202	0.013003	0.012283	0.204689	0.120155	-0.05184	0.125476	0.080315	0.220557	0.112029
C14:0	-0.03776	0.035805	0.172238	0.243839	0.027303	0.008061	0.017529	-0.05846	-0.05035	0.180687	0.110954	-0.09908	-0.11026	0.112719	0.074163	-0.22563	0.074762
C15:0 *iso*	0.029411	0.045056	-0.01293	-0.06662	-0.27038	-0.01931	0.157059	0.079743	0.107765	-0.0775	-0.15124	-0.13572	0.092894	0.06054	0.241479	-0.19564	-0.17382
C15:0 *anteiso*	-0.02819	-0.17131	0.011022	-0.12331	0.042988	-0.06555	-0.08248	0.037994	-0.20921	-0.06572	0.169406	-0.05188	0.579836	-0.0033	-0.08824	-0.16378	-0.12147
C14:1 *cis*-9	0.054807	-0.18645	-0.21673	0.011051	-0.00734	0.054349	-0.06374	0.235237	-0.05636	0.044494	-0.17269	-0.03398	0.056292	0.084086	-0.0817	0.07798	0.000019
C15:0	-0.06193	0.052717	-0.02589	0.042793	0.032054	0.015196	-0.20828	0.104081	0.039279	0.137794	0.028275	-0.22071	0.176332	0.01447	-0.0213	0.028742	0.150164
C16:0 *iso*	0.059919	0.007379	0.181061	0.334751	-0.11529	-0.14428	-0.31698	-0.00571	0.019101	0.078383	-0.1145	0.173617	0.126976	-0.13481	0.3012	0.021329	0.060471
C16:0	0.032313	-0.06452	-0.01385	0.118373	0.030919	0.03001	0.051855	0.113495	-0.04235	-0.24857	-0.08487	0.081367	0.008717	-0.11068	0.036414	0.095372	-0.06328
C17:0 *iso*	0.008031	0.024272	-0.05572	0.071304	0.175585	0.106621	-0.05464	0.064412	0.11741	0.086096	-0.34039	0.114325	0.104886	-0.10244	-0.1906	-0.17768	0.246416
C16:1 *cis*-7	0.186537	-0.11851	0.003345	0.177059	-0.18934	-0.25069	0.338572	-0.21284	-0.22934	0.1318	-0.08244	0.162902	-0.0564	-0.0299	-0.17426	0.006783	0.053393
C16:1 *cis*-9	-0.1304	-0.00196	0.344077	0.039416	0.10206	-0.20554	0.136661	-0.10412	0.171026	-0.18891	-0.12259	-0.09625	-0.09962	0.398811	-0.10866	0.202935	-0.07487
C17:0	0.018929	0.216089	0.156438	-0.12724	-0.01102	0.034394	0.08051	-0.00419	-0.00468	0.081967	0.006994	0.079368	-0.01278	-0.06788	0.033291	-0.01723	0.100613
C17:1 *cis*-9	-0.04666	-0.10935	0.059526	-0.06818	0.213996	-0.20175	-0.15074	0.189927	-0.18131	0.176381	-0.11286	-0.01135	-0.33835	0.022496	0.087857	-0.04755	-0.27437
C18:0	0.034467	-0.03396	-0.23918	0.035413	0.261561	-0.1674	-0.20106	-0.26531	0.374496	0.092953	0.092993	0.041395	0.017942	0.203112	-0.15079	-0.02358	-0.00522
C18:1 *trans*-6[a]	-0.10949	-0.07831	0.152846	-0.04237	0.254897	0.134537	0.036873	0.124605	0.147299	-0.27607	0.137758	0.18256	-0.05477	-0.03927	-0.03044	-0.24735	0.029088
C18:1 *trans*-9	-0.10262	0.026242	-0.13799	-0.08351	-0.02912	0.076672	-0.09429	-0.15855	-0.06262	0.05211	-0.04878	0.052745	-0.01308	-0.08934	0.320003	-0.08613	0.026372
C18:1 *trans*-10	-0.11036	0.063334	0.015597	0.040496	0.241632	0.132588	-0.04238	-0.15701	-0.00247	0.214298	-0.28549	0.00345	-0.07004	0.006577	0.170161	0.177809	-0.1822
C18:1 *trans-11*	0.165947	-0.16653	-0.0561	-0.21642	-0.16581	-0.21291	-0.05092	0.175463	-0.04938	-0.02501	0.104862	-0.10549	-0.164	0.010476	0.203969	0.119456	0.304689
C18:1 *trans*-12	-0.1186	0.065769	-0.18905	0.023256	-0.2096	0.024903	-0.10661	-0.07373	-0.1088	0.000863	0.006477	-0.29618	0.049964	0.088958	-0.21459	0.241846	0.244698
C18:1 *cis*-9	-0.19317	0.08105	0.024925	-0.17783	-0.01199	0.013909	0.020498	0.200034	0.094514	0.050882	-0.03449	0.30646	0.035583	-0.01362	0.0439	0.240041	-0.0459
C18:1 *trans*-15	0.078187	0.230534	-0.05897	0.213102	-0.08932	0.170076	0.113822	-0.09856	-0.06309	-0.00302	0.048083	0.043059	0.134077	0.032264	-0.10618	0.091082	-0.09819
C18:1 *cis*-11	0.010252	0.01993	0.11073	0.067126	-0.10727	0.01609	0.152894	0.021306	0.208239	0.204805	0.12517	-0.17841	0.007982	0.025799	0.060254	-0.13257	0.205249
C18:1 *cis*-12	-0.03392	0.090262	-0.03592	0.063362	-0.0419	0.047983	-0.01805	0.017609	0.104354	-0.1828	-0.02295	0.074555	0.026937	0.029258	0.044642	-0.09431	0.086047
C18:1 *cis*-13	-0.06414	0.172517	0.085538	0.054723	0.141405	0.132254	-0.13386	0.099103	-0.00621	-0.05516	-0.05132	-0.06197	-0.11746	0.015208	-0.00521	0.014352	0.099778
C18:1 *trans-16*[b]	0.007129	0.183089	0.20394	-0.04231	-0.22345	-0.0629	0.00812	-0.20495	0.003493	-0.01704	0.202833	0.00851	-0.12731	-0.0568	0.102763	0.120578	-0.11655
cyclo-17[d]	0.091848	0.126494	0.141639	0.096907	-0.05036	-0.12214	-0.05951	-0.14825	0.110574	-0.03176	-0.08035	0.000369	0.053337	-0.00682	0.051859	-0.05827	-0.14711
C18:1 *cis-16*	-0.32339	0.064928	0.020421	0.111042	0.158479	-0.12108	0.189071	0.09133	-0.14542	0.112097	0.022515	0.032048	0.149538	-0.0689	0.080533	-0.06824	0.054268
C18:2 *trans*-9,*cis-12*	0.152731	0.227785	0.010145	0.0291	-0.04109	0.080049	-0.19496	-0.05672	-0.05188	-0.18377	0.078465	-0.07404	-0.04703	0.157115	-0.05592	0.053475	-0.03643
C18:2 *trans*-11,*cis*-15	-0.0031	0.006766	0.157401	-0.0272	-0.09402	-0.28926	0.134517	0.152918	0.133142	-0.03718	-0.26284	-0.011	-0.03567	-0.03763	-0.30688	-0.18041	0.053827
C18:2 n-6	0.212576	0.136591	-0.06596	-0.10183	0.154946	-0.02941	0.089605	0.043499	0.061998	0.035724	-0.00624	0.009163	0.074894	-0.00099	0.01514	0.087107	-0.04293
C19:1	-0.10165	0.38381	-0.37842	0.173408	-0.09031	-0.18506	0.041653	0.191917	-0.07592	-0.08262	0.06735	0.141168	-0.15356	0.113962	-0.00679	0.033518	0.034119
C20:0	-0.20367	0.267826	0.047464	-0.13304	0.119786	-0.00716	-0.02593	0.035526	0.103585	-0.07487	0.147234	-0.25886	0.038046	-0.0642	0.097506	-0.04363	-0.01488
C18:3 n-6	0.069681	-0.03418	-0.06097	0.211379	-0.03272	0.231206	-0.02382	0.213335	-0.10198	0.100051	-0.13973	-0.17187	-0.04912	0.227667	-0.01041	0.145041	-0.19012
C20:1	-0.01974	-0.1625	0.135405	0.028281	-0.17968	0.100493	-0.07707	0.052799	-0.01422	0.054267	-0.09212	0.304643	0.047098	0.030107	-0.0157	0.196179	-0.03735
C18:3 n-3	-0.09057	-0.02246	-0.02259	0.005069	-0.01217	-0.01382	-0.01766	0.031706	-0.00736	-0.06251	0.001867	-4.4E-05	0.004772	-0.03428	-0.00869	0.061277	-0.02659
C18:2 *cis*-9,*trans*-11	-0.13552	0.10193	0.080532	-0.10291	-0.05922	0.198903	0.126702	-0.03745	-0.23776	0.004643	0.012238	-0.07242	-0.00338	-0.07269	-0.13627	-0.05717	-0.25509
C21:0	0.183286	0.164032	0.098059	-0.013	0.1023	-0.05044	-0.12688	-0.11003	-0.1514	0.055205	0.16411	0.275041	0.097946	-0.12244	-0.01992	0.039609	0.114088
C20:2 n-6	-0.01776	-0.12693	-0.17524	-0.10493	0.093154	-0.27943	0.205457	-0.17159	-0.03965	0.057827	0.010625	-0.13332	0.198635	0.152691	0.225512	-0.03118	-0.13603
C22:0	-0.33867	-0.17017	0.100934	0.25819	-0.10173	-0.04455	0.041172	0.021784	0.212822	-0.10692	0.045992	-0.1281	0.052121	-0.24865	0.019038	0.210263	0.046272
C20:3 n-6	-0.10228	-0.09134	-0.08334	-0.08958	0.146686	-0.08808	-0.04512	-0.09662	-0.15651	-0.17423	0.048504	0.11747	-0.10468	0.046659	-0.06845	-0.04263	0.221642
C20:4 n-6	0.053875	-0.00219	0.096881	-0.14263	0.025297	0.107665	0.03216	-0.07855	-0.02759	0.191617	0.03671	-0.18668	-0.20113	-0.27329	-0.37295	-0.00771	0.029623
C23:0	0.08381	-0.15831	0.005065	-0.05864	0.003753	0.205245	0.002255	-0.11143	-0.01369	0.160048	-0.17419	-0.14806	0.005665	-0.03915	0.135183	-0.14123	0.035263
C24:0	0.010718	-0.14756	-0.02005	0.116853	-0.07473	0.026266	-0.00482	0.028915	0.20479	0.088242	0.02297	0.046822	0.042766	-0.02861	-0.04976	0.076062	0.057772
SFA	0.015032	-0.04622	0.037713	0.16026	0.024806	0.033194	0.0062	0.005459	0.01876	-0.03516	0.068978	0.007343	-0.01708	0.030201	0.025651	-0.08603	-0.02727
MUFA	0.330824	-0.02702	-0.08926	0.375091	0.259853	-0.06982	0.10054	0.004479	0.001211	-0.22773	0.012896	-0.20445	-0.01939	-0.24497	0.052317	0.023026	-0.02737
PUFA	0.200373	0.13558	-0.0661	-0.10917	0.150582	-0.02842	0.092889	0.04635	0.059707	0.03721	-0.00438	0.005738	0.077754	-0.00836	0.007604	0.099505	-0.0466
n-6 PUFA	0.126103	0.042691	-0.01693	0.02384	0.076932	-0.0053	-0.02059	-0.06567	-0.03543	-0.04948	-0.05448	0.014084	-0.08588	-0.03319	-0.01287	-0.07811	-0.113
n-3 PUFA	-0.05051	-0.02087	-0.00483	0.037641	-0.0047	0.000759	-0.0035	0.015935	0.031934	-0.01145	-0.00615	0.000698	0.00005	-0.01837	-0.01659	0.019436	-0.00501
AI	0.122247	0.122271	-0.01558	-0.11794	0.025709	-0.0182	0.115492	0.103619	0.080843	0.107046	0.027107	0.00572	0.076752	-0.01112	0.012339	0.085944	-0.01364
TI	0.126345	0.066873	-0.07574	-0.13587	-0.00722	0.013727	0.045866	0.110582	0.063018	-0.06398	-0.07977	0.105533	0.054106	-0.05209	-0.03461	0.148489	-0.0125
P/S	0.060773	0.046962	0.054764	0.154716	-0.00911	0.065409	0.002234	0.172504	-0.19427	0.085467	0.126675	0.069747	-0.10941	0.329275	0.106089	-0.27311	0.215251
n-6/n-3	-0.05529	-0.06403	-0.00754	0.020907	-0.06692	0.04204	-0.02824	0.012694	0.024932	0.053108	0.000859	0.033234	0.025901	-0.02456	-0.00298	0.012933	-0.0085
PI	0.104711	0.048019	-0.01809	-0.00093	0.077999	-0.00925	-0.0082	-0.05601	-0.02432	-0.03345	-0.03596	-0.01882	-0.07278	-0.04745	-0.02597	-0.07	-0.07946
h/H	-0.09943	-0.03736	-0.02779	-0.00638	-0.1033	-0.01284	-0.20463	-0.15301	0.095334	0.222952	0.080967	0.170907	0.03447	0.299223	-0.19057	-0.10646	-0.13137
C18:2 *cis,trans/trans,cis*[c]	0.197103	0.029316	0.025951	-0.21862	-0.17647	0.058743	-0.1346	-0.08482	0.216772	-0.18456	-0.19479	0.006593	0.016574	0.064273	0.113887	-0.08234	0.062843
CHL	-0.01419	-0.08628	-0.06335	-0.0353	0.067992	-0.07107	-0.03427	0.029407	-0.08372	0.000374	0.016419	0.026923	-0.02068	0.013602	0.010258	-0.00995	0.007608

Variable	PC51	PC52	PC53	PC54	PC55	PC56	PC57	PC58	PC59	PC60	PC61	PC62	PC63	PC64	PC65	PC66
TL	-0.01832	-0.01977	-0.06744	-0.00946	0.020824	-0.00361	0.032049	0.026832	-0.00776	-0.00405	-0.00267	-0.00855	0.019316	-0.00391	-0.00013	-6.4E-05
C4:0	0.025487	0.170624	0.061298	-0.12406	-0.06449	0.022034	0.085072	0.039255	0.001171	-0.00095	-0.01411	0.025092	0.034544	0.004162	0.065263	-0.00787
C6:0	-0.01067	0.02719	-0.02806	-0.06567	-0.036	0.022545	-0.03906	-0.02709	0.038714	0.033939	0.012442	0.010893	-0.02701	-0.00077	0.003854	-0.00044
C8:0	0.179363	0.107301	-0.03394	0.216548	-0.10648	-0.10087	-0.06208	0.070253	0.060795	0.091961	0.054244	-0.02147	-0.11688	-0.01266	-0.00952	0.000724
C10:0	-0.12011	-0.06935	-0.29717	-0.13659	-0.2151	-0.19356	-0.17978	-0.14664	-0.13741	0.014897	0.047099	0.550586	0.227508	0.069757	0.134304	-0.00654
C11:0[a]	0.015301	-0.01406	0.254865	-0.01516	0.096221	0.349464	-0.26454	0.127056	-0.04144	-0.13382	-0.00103	-0.01636	0.026356	0.009515	-0.01315	0.001175
C12:0	-0.12616	-0.07064	-0.13642	-0.25153	0.179991	0.012439	0.336196	-0.12834	0.20445	0.266812	-0.01973	-0.41637	-0.33011	-0.0671	0.036073	-0.01592
C13:0 *anteiso*	0.088445	0.062952	0.216771	-0.02548	-0.0861	0.130727	-0.05641	0.119818	-0.00183	-0.03582	0.012615	0.004201	-0.02494	-0.0002	-0.00395	-0.00099
C12:1	0.196871	-0.14152	-0.19855	0.072376	-0.13623	0.065979	-0.0208	0.004278	0.066211	0.066603	0.02631	0.003614	-0.04577	-0.01059	-0.00252	-0.00124
C13:0	-0.22813	-0.02657	-0.00268	0.042722	-0.00832	0.175297	-0.07416	-0.05075	0.010215	0.003326	-0.01532	-0.02628	0.000329	-0.00418	-0.00025	-0.00073
C14:0 *iso*	-0.33782	-0.28345	0.227189	0.039396	-0.01982	-0.06194	0.035757	-0.07398	-0.052	0.017314	-0.01855	0.019184	0.035615	0.001308	-7.9E-05	0.000481
C14:0	0.047246	-0.04799	0.019326	-0.08878	0.043264	0.306089	0.319749	-0.05078	0.073022	-0.05929	0.161867	0.224757	-0.04383	0.026575	0.377686	-0.05047
C15:0 *iso*	-0.17548	-0.02397	0.226472	-0.04837	0.072749	0.126694	-0.09587	0.02843	-0.00467	-0.10227	0.004196	0.002393	0.05985	0.009045	0.012659	-0.00256
C15:0 *anteiso*	0.106962	0.164513	0.009144	-0.09378	0.003692	0.205414	-0.12014	0.12399	0.043921	0.000342	0.027146	0.037193	-0.05258	0.002928	0.025313	-0.00345
C14:1 *cis*-9	0.051444	0.140218	0.194865	0.352473	0.231772	0.082903	0.317915	-0.33004	-0.39428	0.119176	-0.07656	0.114201	0.116852	-0.00067	0.030352	-0.00012
C15:0	0.020471	-0.40728	0.212502	-0.15937	0.065523	-0.41373	0.22686	0.369786	0.01547	-0.1141	-0.02076	0.017781	0.075433	0.011462	0.004573	-0.00046
C16:0 *iso*	0.188501	-0.07828	-0.02206	0.040322	-0.01402	0.095112	-0.00538	-0.0928	-0.04328	0.09225	0.008321	-0.01144	-0.04638	-0.00928	0.007414	-0.00142
C16:0	-0.03403	0.085315	-0.0476	0.362378	0.096988	-0.09085	-0.0033	0.050156	0.521114	-0.23981	0.002913	-0.13065	0.349759	0.009172	0.346582	-0.04217
C17:0 *iso*	-0.1609	0.129869	0.038998	-0.19862	-0.02926	-0.00107	-0.00838	-0.0559	0.082253	0.029115	0.022509	-0.00963	0.002902	-0.00425	0.012515	-0.00201
C16:1 *cis*-7	-0.00685	-0.10641	0.105014	-0.11568	0.047321	0.036781	-0.01689	0.092216	0.018694	-0.04732	-0.00034	0.011711	0.031412	0.008713	-0.00223	0.000595
C16:1 *cis*-9	0.052377	0.258942	-0.03422	-0.13831	-0.01461	0.043093	0.017687	0.052498	-0.0902	0.013364	0.002021	0.02501	-0.01923	0.005264	0.009609	-0.00036
C17:0	-0.05755	-0.0013	0.051548	0.020532	-0.04176	-0.00818	0.000708	-0.03357	0.024629	0.032793	0.009766	0.018881	0.001986	-0.00207	0.004852	0.000083
C17:1 *cis*-9	0.23504	-0.0011	-0.07739	0.000242	-0.06553	-0.06652	0.015007	0.091458	-0.03141	-0.06611	-0.00849	-0.0283	-0.01694	0.006425	-0.01121	0.001158
C18:0	-0.0329	0.081457	-0.03326	-0.00215	0.254244	0.031038	-0.12822	0.122836	-0.02007	-0.00405	0.002203	-0.03033	0.056239	-0.00172	0.090166	-0.01209
C18:1 *trans*-6[a]	0.396252	-0.15978	0.093329	0.133237	-0.0544	0.03688	-0.05406	0.046671	-0.07216	-0.00868	-0.00772	-0.04672	-0.06287	-0.00939	-0.0168	-0.00142
C18:1 *trans*-9	0.001294	0.412186	0.057302	-0.22781	0.012419	-0.19949	0.199287	0.079714	-0.05282	-0.10925	-0.00765	0.016231	0.090257	0.013838	0.02475	0.001936
C18:1 *trans*-10	-0.27353	-0.12961	-0.17986	0.059416	-0.0162	0.04282	-0.10279	-0.11728	0.026813	0.063048	-0.00392	-0.02649	-0.04078	-0.00322	-0.0148	0.000555
C18:1 *trans-11*	-0.12079	0.151123	-0.10988	-0.04785	-0.22929	0.000917	0.048946	-0.01612	0.153849	-0.00273	0.019552	0.020838	0.004913	-0.00273	0.011284	-0.00107
C18:1 *trans*-12	0.143357	-0.17836	-0.16528	0.082508	0.031733	0.215519	-0.05322	-0.01516	0.153896	0.146126	0.020852	0.075352	-0.0449	-0.00717	0.002473	-0.00224
C18:1 *cis*-9	0.021275	-0.02204	-0.14446	-0.13963	0.35655	0.21315	0.162766	0.254001	0.153111	0.113219	0.017808	0.305444	0.139308	0.037071	0.037176	-5.8E-05
C18:1 *trans*-15	-0.02452	0.156403	0.032759	-0.03015	-0.00924	-0.04392	0.047983	0.006795	-0.10296	-0.01596	-0.0198	-0.01496	-0.00176	0.001795	0.005349	0.000892
C18:1 *cis*-11	0.014855	0.08682	0.00213	0.081584	0.03561	0.014061	0.008452	-0.05588	0.064896	0.041896	0.03998	0.001866	0.008267	-0.01153	0.002027	-8.9E-05
C18:1 *cis*-12	-0.20259	-0.07651	-0.20834	0.122031	-0.19102	0.235122	0.216864	0.282408	-0.30779	-0.18504	-0.12037	-0.1429	0.065338	-0.01332	-0.00179	-0.00335
C18:1 *cis*-13	0.039433	0.095327	0.301288	-0.19994	0.063017	-0.08673	-0.15671	-0.29859	0.226187	0.12576	0.070479	0.10644	-0.03596	0.00804	0.005553	0.000351
C18:1 *trans-16*[b]	0.174606	-0.04222	0.026706	0.162736	0.158006	-0.16705	0.139435	-0.02829	-0.11601	-3.8E-05	-0.01963	0.02837	0.050916	0.005885	0.001131	0.002821
cyclo-17[d]	-0.04158	-0.04647	-0.06881	-0.02317	0.031724	-0.09046	-0.02215	0.010589	0.01373	-0.01519	0.001234	-0.01216	0.002817	0.002078	0.002166	-0.0002
C18:1 *cis-16*	-0.08717	-0.01194	0.014076	-0.01128	-0.08191	-0.0352	0.095683	-0.00161	-0.0389	0.000879	-0.01437	-0.00248	-0.0003	-0.0042	0.00531	0.000684
C18:2 *trans*-9,*cis-12*	0.021654	-0.10741	0.012306	-0.02458	0.029484	-0.05583	0.03278	-0.01366	0.005756	0.019518	-0.00903	0.003588	0.00073	-0.00157	0.001726	-0.00082
C18:2 *trans*-11,*cis*-15	-0.05916	-0.10464	0.026514	0.031329	0.038256	-0.01005	0.006895	-0.02416	-0.02998	-0.01861	-0.0021	0.033808	0.05712	0.003919	0.001535	0.002029
C18:2 n-6	0.120633	-0.11281	-0.06725	-0.16819	0.023429	0.145198	0.148247	-0.1689	0.037786	-0.27089	0.035599	0.021261	0.047618	0.047439	0.098107	0.703319
C19:1	0.093884	0.10083	0.104806	-0.04285	-0.09355	-0.00206	0.001475	-0.01574	-0.01502	-0.00459	0.001039	-0.00135	-0.02558	0.001518	-0.00237	0.00074
C20:0	-0.23295	0.045033	-0.11275	0.167865	-0.03506	0.135492	0.042981	-0.09078	-0.01527	0.01891	-0.01828	-0.04033	0.002793	-0.0202	0.006894	-0.0004
C18:3 n-6	0.093505	-0.00594	-0.10607	-0.08393	-0.18741	0.127426	-0.11469	0.199275	0.036977	-0.05255	0.001309	-0.06667	-0.06483	0.002021	-0.01219	0.001923
C20:1	0.008922	-0.00704	-0.04184	-0.04784	0.006893	-0.04385	-0.03257	0.004168	-0.05154	0.007215	0.004213	-0.01548	0.003135	0.014372	-0.00047	0.00028
C18:3 n-3	-0.00414	-0.02262	0.001823	-0.04286	-0.03111	0.00715	-0.0056	0.039437	-0.11971	0.132241	0.599824	-0.16897	0.239175	-0.1319	-0.00781	0.072234
C18:2 *cis*-9,*trans*-11	-0.01566	-0.09483	0.192086	0.01046	0.149911	0.054352	-0.09985	-0.05212	0.029127	-0.03343	0.006541	0.013527	0.041072	0.009988	0.00096	0.008976
C21:0	-0.00337	0.059443	-0.05881	0.071963	-0.02975	0.060976	-0.00942	-0.03889	-0.02913	0.051979	-0.01264	-0.0203	-0.04378	-0.00735	0.001449	-0.00099
C20:2 n-6	0.100815	-0.06318	-0.13745	0.110637	0.05744	-0.17162	0.007135	-0.10732	0.11166	0.145301	0.041863	0.107161	-0.04935	-0.00166	0.009484	0.003861
C22:0	0.048322	0.073554	-0.02614	-0.04071	0.008217	-0.03663	-0.00611	-0.02794	0.028939	0.016995	-0.01448	0.021274	-0.01735	0.004052	0.006844	-0.00025
C20:3 n-6	0.033337	-0.12855	-0.09633	-0.03634	-0.02519	-0.02369	0.061735	-0.09746	0.045021	0.068424	-0.01388	0.035542	-0.00288	-0.00589	0.002165	0.002409
C20:4 n-6	0.066968	0.252318	-0.18223	0.050152	0.09309	-0.12443	0.1484	0.093322	-0.02041	-0.09041	0.013005	-0.0631	0.045838	-0.03752	-0.00101	0.017648
C23:0	0.091117	-0.06873	-0.06675	0.05692	0.057499	0.024784	0.060887	0.009216	-0.01271	0.014735	0.000185	0.013998	0.025093	-0.00479	0.002903	-0.00098
C24:0	0.016127	0.058994	0.029022	-0.0111	-0.00387	0.007456	-0.01253	-0.02058	-0.00551	0.017214	0.01426	-0.0013	-0.00923	0.000955	0.003321	-0.00031
SFA	-0.02416	0.050522	-0.04707	0.052369	0.065921	0.092504	0.17003	-0.01316	0.248374	-0.07362	0.042323	0.150259	0.186263	0.047622	-0.82421	0.112077
MUFA	0.026411	0.015682	-0.00048	-0.02121	0.016928	0.007612	0.004561	0.008408	-0.00863	0.030689	0.021193	0.01633	-0.00433	-0.00123	-1E-06	-8.1E-05
PUFA	0.12896	-0.10902	-0.07697	-0.17926	0.029117	0.135031	0.143202	-0.18281	0.022402	-0.28628	0.091359	0.02194	0.09293	-0.02722	-0.0899	-0.69225
n-6 PUFA	-0.09423	0.048016	0.151259	0.126721	-0.10035	0.066019	0.140676	0.288237	0.128819	0.32078	-0.04861	0.300499	-0.02743	-0.64124	0.018783	0.009984
n-3 PUFA	0.009568	0.021567	0.002149	0.002339	0.034267	-0.00203	0.007595	-0.03424	0.122201	-0.1775	-0.59504	0.190395	-0.24174	0.015184	0.01509	-0.00046
AI	0.111886	-0.02348	-0.00889	-0.13604	-0.08643	-0.0063	-0.09964	0.002934	-0.02571	0.33559	-0.34002	-0.20591	0.429505	-0.08322	0.018816	0.004116
TI	-0.11354	0.054016	0.022489	0.201097	0.042323	-0.14543	-0.02829	0.142221	-0.00014	-0.13887	0.293631	0.185762	-0.4897	0.061449	-0.02251	-0.00252
P/S	-0.11718	0.072479	-0.18901	0.10361	0.455143	-0.08209	-0.27697	0.109737	-0.03394	-0.02589	-0.00696	-0.079	0.050744	-0.00519	-0.01754	0.001407
n-6/n-3	0.009763	-0.02155	0.018298	0.018661	-0.04191	-0.00821	0.021822	-0.02258	0.028796	-0.00863	0.013381	-0.00684	0.012769	0.001439	0.000513	0.000074
PI	-0.06361	0.039131	0.115356	0.099887	-0.10342	0.070155	0.134517	0.240476	0.080454	0.398387	-0.00049	0.057002	0.034964	0.734642	0.032219	-0.05384
h/H	-0.02364	0.025734	0.195173	0.215394	-0.34846	0.001688	0.142755	-0.12542	0.263062	-0.15809	0.037043	-0.08681	0.103245	-5.9E-05	0.003345	0.000819
C18:2 *cis,trans/trans,cis*[c]	0.088891	-0.08279	0.059903	0.014143	0.071657	0.028239	-0.04633	-0.04171	0.070789	0.060373	0.024012	0.039429	-0.00504	0.000379	0.003608	0.003864
CHL	-0.06496	-0.00658	0.028006	-0.03355	-0.02363	-0.00106	0.004915	0.020133	-0.01346	-0.02295	-0.00931	-0.01023	0.006263	0.001331	-0.00229	-0.00001

[a]C18:1 *trans*-6/*trans*-7/*trans*-8 (co-elution); [b]C18:1 *trans*-16/*cis*-14 (co-elution); [c]Sums of several isomers with unknown geometry; [d]11-Cyclohexyl-11:0; TL= Total lipids; CHL= Total cholesterol.

Table 4 - Principal components loadings of cow's milk from different fat classes (S=skimmed milk; S-S=semi-skimmed milk; W=whole milk) and plant-based milk alternatives (soya, oat, rice, almond, coconut, and hazelnut).

Beverage	PC1	PC2	Beverage	PC1	PC2
S	4.85734	6.6486	Soya	-7.73106	2.6856
S	4.26574	9.9285	Soya	-7.85458	2.7286
S	3.82044	3.9953	Soya	-7.89503	2.6072
S	6.61089	11.0857	Soya	-7.28355	1.3798
S	4.4378	4.02	Soya	-7.80915	2.2431
S	3.20502	0.6047	Soya	-8.30128	1.827
S	5.03367	1.9892	Soya	-7.33474	0.9165
S	4.69503	2.8079	Soya	-8.03629	2.1901
S	4.54521	1.7605	Soya	-7.7321	1.6615
S	3.12532	-0.2445	Soya	-7.75766	1.8415
S	1.72477	-1.0322	Oat	-7.36769	0.5873
S	1.50622	1.0257	Oat	.	.
S	4.40326	3.738	Oat	-7.55007	1.0577
S	3.39958	1.5824	Oat	-7.10325	1.5488
S	4.21128	0.7689	Oat	-6.34682	0.3651
S	4.98963	1.7104	Oat	-7.24242	0.9999
S	3.93762	3.7915	Oat	.	.
S	2.4057	-1.1309	Oat	.	.
S	4.90006	5.8322	Oat	.	.
S	3.82321	1.4758	Oat	-6.79919	0.3679
S-S	4.66704	-1.7051	Rice	.	.
S-S	4.40771	-1.3679	Rice	-7.70917	1.295
S-S	4.24139	-1.2042	Rice	-7.72871	1.7382
S-S	3.99321	-1.143	Rice	.	.
S-S	4.67082	-1.1209	Rice	.	.
S-S	4.75717	-0.6721	Rice	.	.
S-S	3.63505	-0.6207	Rice	.	.
S-S	4.88986	-1.1793	Rice	.	.
S-S	6.01237	-0.7423	Rice	-7.59413	1.2937
S-S	3.91504	-1.5264	Rice	.	.
S-S	4.87609	-1.4089	Almond	-6.91493	-1.5985
S-S	4.51178	-1.1881	Almond	-6.81122	-0.9794
S-S	4.27026	-0.9827	Almond	-6.85429	-1.6198
S-S	4.14142	-1.2789	Almond	.	.
S-S	4.76004	-0.6127	Almond	-6.84608	-1.5435
S-S	4.74805	-1.2257	Almond	-6.72249	-1.688
S-S	4.16271	-1.2717	Almond	-6.85621	-1.801
S-S	4.31725	-1.4756	Almond	-6.84858	-1.7107
S-S	4.71604	-2.032	Almond	-6.67385	-1.7017
S-S	4.35054	-1.4068	Almond	-6.59051	-1.6788
W	4.56136	-1.6544	Coconut	-2.96001	-1.1891
W	4.81912	-1.7785	Coconut	-3.60146	-0.8331
W	6.26436	1.1538	Coconut	-3.66761	-0.6546
W	4.33692	-1.7218	Coconut	-3.49015	-0.775
W	4.74291	-1.5999	Coconut	-2.93949	-1.2531
W	4.49298	-1.5825	Coconut	-3.12746	-1.281
W	6.82866	-1.8424	Coconut	.	.
W	4.69078	-1.962	Coconut	-2.70555	-1.9299
W	4.40276	-2.1114	Coconut	.	.
W	4.64233	-1.7607	Coconut	.	.
W	4.7727	-1.8471	Hazelnut	.	.
W	4.82857	-1.4588	Hazelnut	.	.
W	4.81963	-1.6982	Hazelnut	.	.
W	4.43751	-2.1237	Hazelnut	-6.38332	-1.8461
W	5.17019	-1.1874	Hazelnut	-6.47447	-1.9812
W	4.4887	-1.8859	Hazelnut	-6.53763	-1.7997
W	4.12931	-1.1889	Hazelnut	.	.
W	4.49964	-2.0379	Hazelnut	-6.57415	-1.7963
W	4.38712	-2.1362	Hazelnut	-6.60748	-1.9102
W	4.75121	-2.0587	Hazelnut	-6.64459	-1.4738
